# Comparative mapping in the Fagaceae and beyond with EST-SSRs

**DOI:** 10.1186/1471-2229-12-153

**Published:** 2012-08-29

**Authors:** Catherine Bodénès, Emilie Chancerel, Oliver Gailing, Giovanni G Vendramin, Francesca Bagnoli, Jerome Durand, Pablo G Goicoechea, Carolina Soliani, Fiorella Villani, Claudia Mattioni, Hans Peter Koelewijn, Florent Murat, Jerome Salse, Guy Roussel, Christophe Boury, Florian Alberto, Antoine Kremer, Christophe Plomion

**Affiliations:** 1 INRA, UMR1202 BIOGECO, Cestas, F-33610, France; 2 Université de Bordeaux, UMR1202 BIOGECO, Cestas, F-33610, France; 3 Forest Genetics and Forest Tree Breeding Büsgen Institute Faculty of Forest Sciences and Forest Ecology Göttingen University, Büsgenweg 2, 37077, Göttingen, Germany; 4 Plant Genetics Institute, National Research Council, Via Madonna del Piano 10, Sesto Fiorentino, FI, 50019, Italy; 5 Plant Protection Institute, National Research Council, Via Madonna del Piano 10, Sesto Fiorentino, FI, 50019, Italy; 6 NEIKER-Tecnalia, Dpto Biotecnologia, PO Box 46, Vitoria-Gasteiz, 01080, Spain; 7 Unidad de Genética Ecológica y Mejoramiento Forestal, INTA EEA Bariloche, Bariloche, CC277 8400, Argentina; 8 CNR Istituto di Biologia Agroambientale e Forestale, Porano, TR, 05010, Italy; 9 ALTERRA Wageningen UR, PO Box 47, Wageningen, 6700 AA, The Netherlands; 10 INRA, UMR1095 GDEC, Clermont-Ferrand, F-63100, France; 11 New address: School of Forest Resources and Environmental Science, Michigan Technological University, Houghton, MI, 49931, USA

## Abstract

**Background:**

Genetic markers and linkage mapping are basic prerequisites for comparative genetic analyses, QTL detection and map-based cloning. A large number of mapping populations have been developed for oak, but few gene-based markers are available for constructing integrated genetic linkage maps and comparing gene order and QTL location across related species.

**Results:**

We developed a set of 573 expressed sequence tag-derived simple sequence repeats (EST-SSRs) and located 397 markers (EST-SSRs and genomic SSRs) on the 12 oak chromosomes (2n = 2x = 24) on the basis of Mendelian segregation patterns in 5 full-sib mapping pedigrees of two species: *Quercus robur* (pedunculate oak) and *Quercus petraea* (sessile oak). Consensus maps for the two species were constructed and aligned. They showed a high degree of macrosynteny between these two sympatric European oaks. We assessed the transferability of EST-SSRs to other Fagaceae genera and a subset of these markers was mapped in *Castanea sativa*, the European chestnut. Reasonably high levels of macrosynteny were observed between oak and chestnut. We also obtained diversity statistics for a subset of EST-SSRs, to support further population genetic analyses with gene-based markers. Finally, based on the orthologous relationships between the oak, *Arabidopsis*, grape, poplar, *Medicago*, and soybean genomes and the paralogous relationships between the 12 oak chromosomes, we propose an evolutionary scenario of the 12 oak chromosomes from the eudicot ancestral karyotype.

**Conclusions:**

This study provides map locations for a large set of EST-SSRs in two oak species of recognized biological importance in natural ecosystems. This first step toward the construction of a gene-based linkage map will facilitate the assignment of future genome scaffolds to pseudo-chromosomes. This study also provides an indication of the potential utility of new gene-based markers for population genetics and comparative mapping within and beyond the Fagaceae.

## Background

Genetic linkage maps constitute an ideal framework for studies of the genetic architecture of quantitative traits [1,2] and genome evolution [3,4]. They are also a prerequisite for map-based gene cloning [5-7] and for the ordering of physical scaffolds in genome sequencing projects [8]. Furthermore they are essential tools for marker assisted plant breeding [9].

Comparative analyses of genetic maps across phylogenetically related species are based on the development of transferable and orthologous genetic makers. Simple sequence repeats (SSRs) are the markers of choice, because they are reproducible, abundant in the genome and they provide highly polymorphic information and are readily transferable between phylogenetically related species [10]. Their properties are highly prevalent in EST-derived SSRs, making these markers particularly useful, as shown for *Theobroma*[11], *Silena*[12], *Prunus*[13], *Dactylis*[14] and *Citrus*[15]. SSRs are also easy to handle and, once developed, are cost-effective markers for high-throughput genotyping.

In the last 12 years, several linkage maps have been generated for the three main genera of the Fagaceae family: oaks (*Quercus*), beeches (*Fagus*), and chestnuts (*Castanea*). These long-lived species constitute important economic and ecological resources and have been the focus of genetic investigations relating to their evolution and more applied objectives, such as those of conservation and breeding programs [16]. Linkage maps have been established to support forward genetic approaches for studying the genetic architecture of adaptive traits (number, location and effect of QTLs) and to increase our knowledge of the structural features of the oak genome and its evolutionary history.

First-generation linkage maps have been obtained with anonymous RAPD and AFLP markers for oak [17], chestnut [18,19] and beech [20]. QTL studies, mostly in oak, have focused on dissecting the genetic architecture of adaptive traits, such as growth and bud phenology [21-23] and of traits related to species divergence between pedunculate and sessile oaks, two species occurring in sympatry in Europe [24,25]. A limited number of genomic SSRs (about 50) and EST-based (about 50) markers [26,27], have also been added to these maps. These markers allowed to align homologous linkage groups between oak and chestnut and to compare and validate the QTLs that had been previously characterized in the two genera [27,28]. A first step toward the construction of a dense SSR-based genetic map was taken recently, with the development and mapping of 256 EST-SSRs [29]. The authors used a selective mapping strategy with a bin set of 14 highly informative offspring from a single full-sib (FS) mapping population for which an AFLP framework map was available. SSR markers were assigned to 44 bins of the female and 37 bins of the male parental maps, spanning the entire genome.

The main goal of this study was to advance the establishment of a dense EST-SSR-based map for oak, by genotyping trees with a broader genetic background and using a larger set of genomic and EST-SSRs. Our specific objectives were as follows:

i) To optimize comparative mapping between two *Quercus* species by identifying a subset of SSRs that were transferable and orthologous across different mapping pedigrees. We genotyped a total of 400 offspring from five families obtained from controlled crosses of the *Q. robur* and *Q. petraea* genotypes. We then generated 10 individual linkage maps (one for each of the parents used in the crosses) by the two-way pseudo-testcross mapping strategy [30] and constructed consensus maps for each species from 419 genomic and EST-based SSR markers.

ii) To determine gene content (synteny) and order (collinearity) between these two sympatric species [7,30-32].

iii) To assess the transferability of a subset of EST-SSRs in several Fagaceae and Nothofagaeae species and to describe the genetic diversity of several oak populations depending on the type of the repeated motifs. We also mapped transferable EST-SSRs, in European chestnut for which two linkage maps were available [28] making it possible to refine the first comparative map for oak and chestnut [27].

iv) To unravel the evolutionary paleohistory of oak chromosomes, by genetic mapping of 321 EST-SSR and 60 SNP-based markers identified from oak transcriptome sequence information (31,798 Sanger-based unigenes from Ueno et al. [33]).

**Figure 1:**
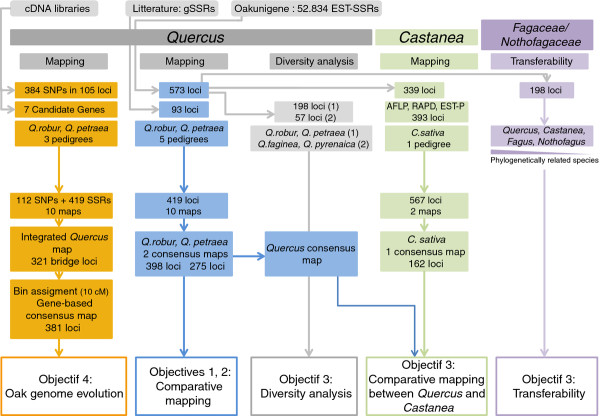
**Relationships between the four objectives of this study.** Inputs, outputs and species involved.

These four objectives are interconnected, as shown in Figure 1.

## Methods

### Functional annotation of EST-SSRs

The functional annotation of EST-SSRs was based on Gene Ontology [34] and was performed with Blast2GO [35], using the following parameters: Blastx search against the non redundant NCBI database (e-value of 1e^-6^).

On the basis of GO categories, we assigned oak ESTs containing SSR motifs (Ueno et al. [33]) to three principal groups: biological processes, cellular compounds and molecular functions. The GO classification obtained was compared (with Expander software, [36]) between four sets of sequences containing SSRs: 3’UTRs (7,680 elements), 5’UTRs (8,646 elements), coding regions (13,899 elements) and non-coding regions (15,829 elements).

### Mapping of SSRs in *Q. robur* and *Q. petraea* and construction of consensus species maps

#### Mapping populations

Five mapping pedigrees (P1-P5) of variable sample sizes were used (Table 1), consisting of one *Q. robur x Q. petraea,* one *Q. petraea* and three *Q. robur* full-sib families. These full-sibs were installed at the nurseries of INRA (Cestas-France), the University of Göttingen (Germany) and Alterra (Wageningen -The Netherlands). DNA was extracted from the leaves with the DNeasy plant mini kit (Qiagen, Hilden, Germany), according to the manufacturer’s instructions.

**Table 1:** Description of the five full-sib mapping populations (N: sample size used for linkage mapping)

	**Short name**	**Species**	**N**	**Location**	**Country**
3P x A4	P1	*Q. robur* x *Q. robur*	92	44°44 N, 0.46°W	France
11P x QS29	P2	*Q. robur* x *Q. petraea*	84	44°44 N, 0.46°W	France
QS21 x QS28	P3	*Q. petraea* x *Q. petraea*	78	44°44 N, 0.46°W	France
SL03 x EF03	P4	*Q. robur* x *Q. robur*	101	51°53'N, 9°93'E	Germany
AltP1 x AltP2	P5	*Q. robur* x *Q. robur*	96	51°98'N, 5°66'E	The Netherlands

#### Development of SSR markers and genotyping

A subset of 573 EST-SSRs identified by Durand et al. [29] was screened for polymorphisms against the 10 parents of the five mapping populations and four offspring per pedigree. We added 93 genomic SSRs (gSSRs) described in previous studies [37-44], KawaharaT pers comm) to the screening step (see Additional file 1 for detailed information on markers and primer sequences). We then used the polymorphic markers to genotype the five mapping populations.

PCR amplification and fragment separation were optimized for M13 fluorescently labeled tailed primers [44]. PCR was performed in a final volume of 10 μL containing 1 x PCR buffer [10 mM Tris–HCl, 50 mM KCl 1.5 mM MgCl_2_, pH 8.3 at 25°C] (BioLabs, Ipswich, England), 100 μM of dNTPs, 0.045 μM forward primers, 0.165 μM reverse primer (5 μM), 0.165 μM M13 primer, 0.25 U of *Taq* polymerase (BioLabs) and 5 to 10 ng of plant DNA. The cycling conditions were as described by Schuelke et al., [45]: initial denaturation at 94°C for 4 minutes, followed by 35 cycles of 94°C for 30 s, 56°C for 45 s, and 72°C for 45 s, nine touchdown cycles of 94°C for 30 s, 53°C for 45 s, and 72°C for 45 s and a final extension at 72°C for 10 minutes. Depending on the pedigree and partners, electrophoresis was performed with the Licor 4200 IR2 system (Lincoln, NB, USA), the ABI 3100 system (Applied Biosystems, Carlsbad, CA, USA) or the Megabace TM 1000 96 capillary electrophoresis system (GE Healthcare, Buckinghamshire, UK). The data generated were analyzed with the 4300 DNA analyzer software for the Licor system, GeneScan 3.7 and Genotyper 3.7 for ABI 3100 and Fragment profiler1.2 for Megabace.

#### Individual map construction

We constructed 10 parental genetic linkage maps (7 for *Q. robur* and 3 for *Q. petraea*) by the two-way pseudo-test cross mapping strategy [30]. Linkage analysis was performed with JoinMap version 4.0 [46]. Polymorphic SSR loci were classified into three categories: testcross markers segregating in a 1:1 ratio, testcross markers segregating in a 1:1:1:1 ratio and intercross markers segregating in a 1:2:1 ratio. Chi-squared goodness-of-fit tests were used to identify markers with patterns of segregation departing from Mendelian expectations. Loci with distorted ratios (*P*-value <0.05) were excluded from linkage map construction. Individuals and loci for which more than 50% of the data were missing were excluded from the analysis. A minimum LOD score of 3 and a maximum recombination fraction of 0.45 were set as the linkage thresholds for marker grouping. Maternal and paternal datasets were created with the “create maternal and paternal population nodes” command in JoinMap. The regression mapping algorithm was used for map construction. Recombination frequencies were converted into map distances in centimorgans (cM), with the Kosambi mapping function. Linkage groups were drawn with MapChart [47].

#### Estimation of genome size

Genome length (L) was estimated from partial linkage data, according to the formula L = n(n-1)d/k, where n is the number of framework markers, d is the maximum distance between two adjacent markers (in cM) at a minimum LOD score for linkage, and k is the number of marker pairs with a LOD value exceeding a minimum threshold [48,49]. LOD score thresholds of 3, 4 and 5 were used to estimate genome length.

#### Construction of consensus genetic linkage maps for Q. robur and Q. petraea

Consensus species maps for *Q. robur* and *Q. petraea* were established by combining parental map datasets for *each* species with the “join-combine groups for map integration” command of JoinMap, which creates a composite map from different linkage groups sharing common markers. We used the mapping parameters and options described above. We assessed the heterogeneity of recombination rates between SSR marker pairs, and SSR markers with highly heterogeneous recombination rates were excluded from the construction of species-specific framework maps. Markers that could not be ordered with the same degree of confidence were added as accessory markers, using the two-point LOD scores and recombination fraction available from the “maximum linkage” table of JoinMap. Similarly, when several markers were found to be collocated, only one was retained on the species framework map; the others were added as accessory markers.

#### Databases

Single-tree genotypic data for offspring and linkage maps are available from the QuercusMap database of the *Quercus* portal (https://w3.pierroton.inra.fr/QuercusPortal/index.php), the European genetic and genomic web resources for *Quercus*. DNA sequences and primer pairs for SSR loci are available from the SSR database at the same URL.

### Transferability of EST-SSRs and comparative mapping of oak and chestnut

#### Transferability of EST-SSRs

We assessed the transferability of EST-SSR markers to other Fagaceae species, by carrying out cross-species amplification in six species (*Castanea sativa*, *Fagus sylvatica*, *Quercus faginea, Quercus pyrenaica, Quercus ilex,* and *Quercus suber*). We also assessed transferability to two species of the related family Nothofagaceae (*Nothofagus pumilio* and *Nothofagus antarctica*). Each species was represented by at least two individuals. SSR amplification and genotyping were performed as described above, with a subset of 243 EST-SSRs randomly selected from the list reported by Durand et al. [29]. The selected microsatellite markers included 137 di-, 90 tri-, 2 tetra-, 1 penta- and 13 hexanucleotide repeats.

#### Comparative mapping of Quercus and Castanea

In total, 96 offspring of a single full-sib pedigree of *Castanea sativa* were genotyped with *Quercus* EST-SSRs. We assessed the amplification of 339 loci using the PCR conditions described above. Polymorphic SSRs were added to the polymorphic markers already available for this pedigree (including RAPD, AFLP, gSSR, EST-P markers; 393 loci in total). Individual parental maps and a consensus map were constructed with JoinMap, using the same procedure followed for *Quercus*. Finally, homologous linkage groups in *Quercus* and *Castanea* were identified from the location of orthologous markers displaying multiple and parallel linkages.

### Diversity analysis

Two experiments were carried out to provide insight into the genetic diversity of EST-SSRs. The first focused on a large number of loci in a small number of individuals of the two sympatric species *Q. robur* and *Q. petraea* (Additional file 2). We assessed the polymorphism of the same set of 243 oak EST-SSRs for 12 individuals from each species. DNA was extracted from leaves with the DNeasy plant mini kit (Qiagen). An M13 tail (TGT AAA ACG ACG GCC AGT) was added to the 5’-end of each forward primer, as described by Schuelke [45]. Each PCR was performed in a total volume of 11 μL containing 1 x PCR buffer, 200 μM of each dNTP, 0.5 U *Taq* polymerase (XtraTaq, Genespin, Milan, Italy), 1.5 mM MgCl_2_, 0.2 μM of each primer, and 10 ng of template DNA. All EST-SSR markers were amplified with an Eppendorf thermal cycler (Mastercycler, Hamburg, Germany), by a touchdown procedure: 3 min at 94°C, 10 touchdown cycles of 94°C for 30 s, 60°C for 30 s (−1°C/cycle), 72°C for 30 s; 27 cycles of 94°C for 30 s, 50°C for 30 s, 72°C for 30 s and a final extension at 72°C for 10 min. The fluorescently labeled PCR products were separated by capillary electrophoresis, with a 400 bp size standard, in a Megabace TM 1000 96 capillary electrophoresis system. Alleles were sized with Fragment profiler version 1.2.

Genetic diversity parameters (AR, H_o_, H_e_) of *Q. robur* and *Q. petraea* were calculated using the FSTAT software package version 2.9.3 [50], which is implemented for the sample-size independent rarefaction analysis of allelic richness.

The second experiment was conceived as a proof of concept for the use of the EST-SSRs in the genetic analysis of the largely unknown semi-decidious oak species distributed around the Mediterranean basin. We genotyped 96 individuals from the two sub-mediterranean oak species *Quercus faginea* and *Quercus pyrenaica* with 64 EST-SSRs evenly distributed among the 12 linkage groups (25 in common with the previous experiment). Additional file 3 shows the locations of the 8 populations per species that were selected to represent most of the geographic and ecological variation in the two species. DNA was extracted from leaf samples using a modified CTAB method because many of the *Q. pyrenaica* samples clogged the columns of commercial DNA extraction kits. The main modification to the standard DNA extraction procedure was the thorough chloroform extraction (3–4 times with a 1:1 volume) following cell lysis in the CTAB buffer. PCRs were performed in a total volume of 10 μL containing 1 x PCR buffer, 100 μM of each dNTP, 0.25 U *Taq* polymerase (KAPA Taq, KapaBiosystems, Boston, USA), 2 mM MgCl_2_, 0.045 μM forward primers, 0.165 μM reverse primer, 0.165 μM M13-fluorescent primer and 10 ng of template DNA. Cycling conditions consisted of an initial denaturation step (94°C 5 minutes) followed by 7 touch-down cycles (from 63.7 to 59.5°C), 20 cycles at 59.5°C annealing temperature, 12 cycles at 57.5°C annealing temperature and a final extension step (10 minutes at 72°C). The fluorescently labelled PCR products were electrophoretically separated in an ABI3130 sequencer (Applied Biosystems) using the GS500LIZ size standard. Peak sizes were scored with GeneMapper v.4.0 and allele binning was performed with MsatAllele R package [51]. Genetic diversity parameters (AR, H_o_, H_e_) were estimated with Fstat v.2.9.3 [50].

#### Oak genome evolution

##### Gene choice and genotyping of SNP-based markers

We constructed an integrated map for *Quercus* based on the EST-SSRs and an additional set of SNP-based markers, for analysis of the synteny between the oak linkage map and those of other previously sequenced eudicots.

We identified 105 candidate genes (set 1) for involvement in bud burst on the basis of the following criteria: i) differential expression between the periods before and after bud flush [52], ii) colocalization with bud burst QTLs [22], and iii) a known functional role in model plants. Two types of polymorphisms were identified: *in vitro* SNPs/Indels from resequenced gene fragments from a panel of nine oak populations [22] and unpublished data and *in-silico* SNPs/Indels retrieved from expressed sequence tags [33] as described by Lepoittevin et al. [53]. Finally, 78 *in vitro* and 306 *in silico* SNPs/Indels were included in a 384-SNP assay, including 26 insertions-deletions (indels) of between 1 and to 3 bp in size. Full description of the SNP array is provided by Alberto et al. [54].

Genotyping was carried out on three mapping populations comprising 177, 80 and 90 F1 plants from the P1, P2 and P3 pedigrees, respectively. DNA was extracted with the *Invisorb DNA plants 96 kit* from Invitek (GmbH, Berlin, Germany), according to the manufacturer’s instructions. Multiplex reactions were prepared with 250 ng of template DNA per sample. Genotyping was carried out with the Illumina GoldenGate SNP genotyping platform (Illumina, San Diego, CA, USA) at the Genome-Transcriptome Facility in Bordeaux, France (http://www4.bordeaux-aquitaine.inra.fr/pgtb). The intensity of the fluorescent signals was measured with the BeadXpress Reader (Illumina Inc, San Diego, USA) and analyzed with GenomeStudio v 3.1.14 (Illumina Inc). Quality scores were generated for each genotype, using a GenCall50 (GC50) score cutoff of 0.25 and a CallRate (CR) threshold of 0.85. These scores reflect the quality of genotype clusters (GC50) and the proportion of samples with a genotype defined for a particular SNP (CR) [55]. Genotype clusters were adjusted manually if necessary.

In addition to this first set of markers, seven candidate genes (set 2) for drought and hypoxia tolerance (from 25 genes initially screened) were found to be informative in one to three pedigrees. Two methods were used for genotyping: i) SSCP (single-strand conformation polymorphism [56], which was used for the first time on a Licor sequencer (see Additional file 4), and ii) primer extension with the detection of fluorescence polarization [57] with the Acycloprime-FP SNP detection kit (Perkin Elmer Life Sciences, Boston, MA, USA). Genotyping was carried out in accordance with the kit manufacturer’s instructions and fluorescence was measured with a fluorescence polarization reader (Victor-Wallace from Perkin Elmer Life Sciences, Boston, MA, USA) at 20, 25 and 35 cycles.

#### Consensus map construction

The consensus map was constructed by joining the 10 independent parental maps based on the mapping populations in which EST-SSRs (P1 to P5) and SNP-based markers (P1 to P3) were mapped. JoinMap was first used to calculate individual maps from raw segregation data, with the Kosambi mapping function. A minimum LOD score threshold of 3.0 was used for the grouping of all markers. An integrated map was then constructed for each linkage group, by integrating the “bridge markers” common to two or more individual maps. For construction of the consensus map, we assumed that the rates of recombination between the two species and between male and female maps were uniform (but see [58]).

Using the regression algorithm of JoinMap, we obtained three maps with different levels of statistical support for ordering (denoted map1-map2-map3 in descending order of statistical support) for each linkage group. For macrosynteny analysis, we decided to retain the most reliable map (map1), adding markers with lower LOD scores as accessory markers. The position of each accessory marker relative to its most probable framework marker was then determined from the two-point LOD scores and recombination fractions provided by the “maximum linkage” table of JoinMap. Finally, markers were assigned to 10 cM bins within each of the 12 linkage groups of the consensus map, for the identification of regions orthologous to sequences in *Arabidopsis*, grape, poplar, *Medicago* and soybean.

#### Evolutionary analysis

##### Genome sequences

The *Arabidopsis* (5 chromosomes - 33198 genes - 119 Mb - ftp://ftp.arabidopsis.org/home/tair/Genes/TAIR9_genome_release/TAIR9_sequences/), grape (19 chromosomes - 21189 genes - 302 Mb - http://www.genoscope.cns.fr/externe/Download/Projets/Projet_ML/data/), poplar (19 chromosomes - 30260 genes - 307 Mb - ftp://ftp.jgi-psf.org/pub/JGI_data/Poplar/), *Medicago* (8 chromosomes - 38834 genes - 261 Mb - ftp://ftpmips.helmholtz-muenchen.de/plants/medicago/) and soybean (20 chromosomes - 46194 genes - 949 Mb - ftp://ftp.jgi-psf.org/pub/JGI_data/phytozome/v5.0/Gmax/) genome sequences were downloaded. CDS annotations (identity, sequence, position) were considered for the synteny and duplication analyses described below. We mined the *Arabidopsis*, grape, poplar, *Medicago* and soybean sequence databases to identify genes paralogous and orthologous to the 31,798 Sanger-based oak unigenes described by Ueno et al. [33].

#### Synteny and duplication analysis

We used BLAST to align genomes (*i.e.* CDS for sequenced genomes and ESTs for oak). We used two parameters for these analyses, to take into account not only similarity but also the relative lengths of the aligned sequences: CIP (cumulative identity percentage) and CALP (cumulative alignment length percentage). CIP = ∑[ID x (HSP/AL) x 100] corresponds to the cumulative percentage sequence identity observed for all the high-scoring sequence pairs (HSPs) divided by the cumulative aligned length (AL), which corresponds to the sum of all HSP lengths. CALP [AL/Query length], is the cumulative AL for all HSPs divided by the length of the query sequence. The use of these parameters for BLAST analysis resulted in the highest cumulative percentage identity over the longest cumulative length, thus maximizing stringency in the definition of conservation between the two genomes compared.

#### Distribution of paralogous and orthologous gene pairs

We estimated sequence divergence and dated speciation events, based on the rates of non-synonymous (*Ka*) and synonymous (*Ks*) substitutions calculated with MEGA-3 [59]. The mean substitution rate (r) for grasses — 6.5 × 10^-9^ substitutions per synonymous site per year — was used to determine the ages of the genes considered [60,61]. The time (*T*) since gene insertion was then estimated with the formula *T* = *Ks*/r.

## Results

### Functional annotation *of EST-SSRs*

We identified more than 52,834 EST-SSRs among the *Quercus* ESTs [33]. As a first step towards functional characterization of the EST-containing SSRs, we used the Slim GO classification and compared the annotations for four sets of sequences containing SSRs: coding regions (CRs), non-coding regions (NCRs), 5’UTRs and 3’UTRs. We identified 35, 7 and 19 gene categories, at “level 3”, within the biological process (BP), cell compound (CC) and molecular function (MF) classes, respectively (Additional file 4). About half the SSRs in the BP class belong to four main categories: “primary metabolic processes” (11%-13.6%), “cellular metabolic processes” (11.7%-14.8%), “macromolecule metabolic processes” (8.1%-10.6%) and “biosynthetic processes” (7.7%-9.9%). For the CC class, 80% of the SSRs were assigned to the “cell part” (42.5%-44.8%) and “membrane-bound organelle” (30.8%-32.3%) categories. For the MF class, six categories of similar size accounted for most of the SSRs: namely “nucleic acid binding” (10.7-14.4), “protein binding” (12.5-13.5%), “nucleotide binding” (10.5%-11.1%), “ion binding” (10.1%-10.6%), “transferase activity” (9.8%-11.3%) and “hydrolase activity” (9.7%-10.1%). The distribution of these categories was similar between the four datasets (Additional file 5), indicating a lack of ability of gene ontology to discriminate between the different transcribed regions in terms of the presence of SSRs. However, slight differences between transcribed regions were nevertheless observed when all categories were considered together in a hierarchical clustering analysis (Additional file 6), but the distribution between the BP, CC and MF classes of the four datasets remained inconsistent.

### SSR-based map construction in *Q. robur and Q. petraea* and synteny analysis

#### Identification of polymorphic markers

In total, we identified 573 primer pairs, which were tested for polymorphism in at least one pedigree (Table 2). Overall, 378 EST-SSRs were informative. We tested 93 of the gSSRs already available for the Fagaceae; 68 (73%) were found to be polymorphic in at least one pedigree. Thus, in total, 446 polymorphic loci (68 gSSRs and 378 EST-SSRs) were available for further mapping.

**Table 2:** Summary of polymorphism statistics for the five pedigrees (P1 to P5), Na not available

	**P1**		**P2**		**P3**		**P4**		**P5**	
tested loci	321		434		406		Na		Na	
polymorphic loci	274 (85%)		211 (49%)		243 (60%)		145 (% Na)		143 (% Na)	
marker type 1:1:1:1	133		110		130		Na		70	
1:1	134		96		100		Na		63	
1:2:1	7		5		13		Na		10	
EST-SSR	229		167		217		122		125	
g-SSR	45		44		26		23		18	
	female	male	female	male	female	male	female	male	female	male
genotyped offspring	46		84		78		96		101	
discarded offspring	0	0	6	4	0	1	3	4	10	11
polymorphic loci	**205**	**195**	**169**	**144**	**190**	**171**	**110**	**114**	**100**	**103**
discarded loci	0.5% (1)	0% (0)	0.6% (1)	0.7% (1)	0% (0)	0% (0)	2% (2)	0% (0)	20% (20)	15% (16)
distorded	2.4% (5)	1.5% (3)	1.8% (3)	6.9% (10)	2.1% (4)	1.8% (3)	2.7% (3)	0.9% (1)	2% (2)	3.9% (4)
unlinked loci	4.3% (9)	6% (12)	4% (7)	7.6% (11)	0.5% (1)	0.6% (1)	0% (0)	0.9% (1)	23% (23)	25% (26)
mapped loci	93% (190)	93% (180)	933% (158)	85% (122)	975% (185)	98% (167)	95% (105)	98% (112)	55% (55)	55% (57)

#### Construction of individual linkage maps

Genotypic data were available for 446 loci in one to five mapping populations among them 397 were mapped. We found that 50% to 85% of the loci tested were polymorphic, depending on the pedigree. The interspecific pedigree (P2) was found to be less polymorphic than the intraspecific pedigrees (Table 2). Differences in levels of polymorphism between intra vs. interspecific pedigrees are likely be due to sampling effects, as the two species exhibit similar levels of genetic diversity and very low interspecific differentiation. The number of tested loci found to be polymorphic varied considerably between the parents, from 100 loci for P5-female (*Q. robur*) to 205 loci for P1-female (*Q. robur*). Distorted loci were more frequent for P2-female (*Q. robur*) from the interspecific pedigree (6.8%) than for the other pedigrees (Table 2). Unlinked loci were rare (except for both of the parental maps for P5, in which 45% of the loci were ungrouped). In the following analyses, we focused on the four pedigrees (P1 to P4) because of the smaller set of data for P5.

##### Linkage group (LG) statistics

We constructed 12 LGs for each parental map, except for the interspecific P2-male parent, for which LG11 was missing due to the fact that all markers were distorted and therefore excluded a priori from linkage map construction. Interestingly, LG11 for the P2-female parent also included four distorted loci suggesting the presence of loci involved in species incompatibility. The development and mapping of a large amount of SNP markers will certainly provide new insights into the identification and mapping of loci involved in reproductive barriers between these two hybridizing oak species.

The mean number of markers per LG was between 7.6 for LG4 and 26.9 for LG2 over the 8 maps, (“groups” with only 2 markers were not considered) (Additional file 7). The mean map length of the various LGs was between 42.2 cM for LG4 and 85.3 cM for LG2, with an overall mean of 58.4 cM over the 8 maps (Additional file 8).

##### Estimation of total genome length

The total observed map length was 572.9 cM to 846.6 cM (Additional file 8). Based on these partial linkage data, estimated genome sizes were obtained for various LOD score values (3, 4 and 5). They ranged from 945 to 1,611 cM (Additional file 9).

##### Number of alleles of mapped SSRs

The number of alleles observed in the 10 parents depended on the type of motif considered: the number of loci with three or four alleles was systematically higher for loci with dinucleotide repeats than for those with trinucleotide or hexanucleotide repeats (Table 3). This trend was conserved even if we excluded gSSRs from the analysis.

**Table 3:** Type of SSR motifs for the five pedigrees

**Motif type**	**Number of loci/motif type**	**Number of alleles**		
		**2**	**3**	**4**
di	503	200 (0.4%)	132 (0.26%)	171 (0.34%)
tri	259	163 (0.63%)	58 (0.22%)	38 (0.15%)
hexa	59	16 (0.27%)	4 (0.07%)	1 (0.02%)

##### Mapping with several pedigrees

**Figure 2:**
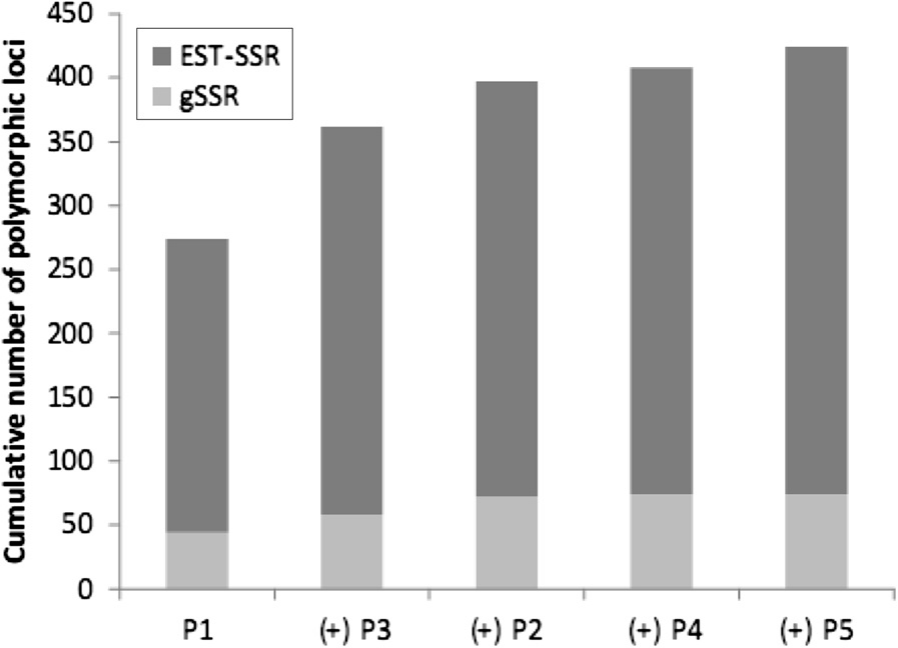
Information gained by genotyping several pedigrees (P1 to P5).

**Figure 3:**
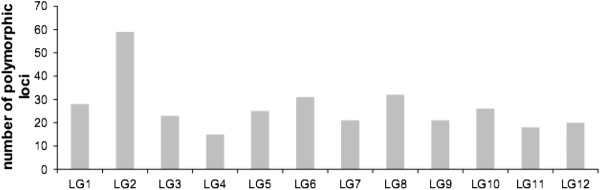
Distribution of common loci per LG for at least two maps.

**Figure 4:**
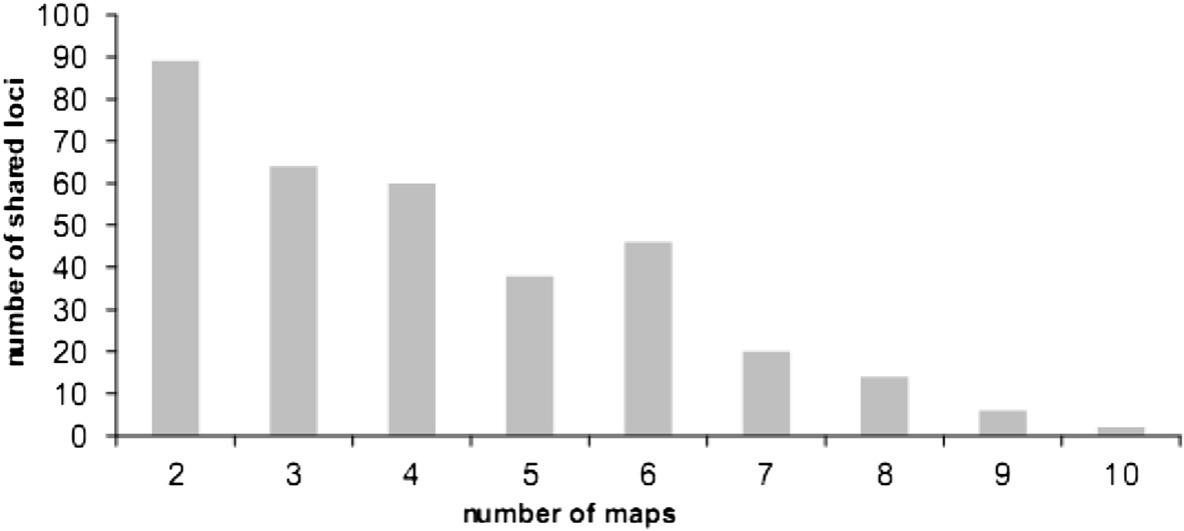
Number of shared loci between two to ten parental maps.

The genotyping of several pedigrees significantly increased the number of loci identified as polymorphic (Figure 2). P1 was the most informative pedigree in terms of mapped markers (274 polymorphic loci). However, adding P3 to the analysis increased the number of markers identified as polymorphic by 24% (88 new markers). Successive additional inclusions of P2, P4 and P5 increased the number of polymorphic markers identified by 35, 11 and 16 SSRs, respectively. For the 12 linkage groups obtained for each parental map, the number of markers common to at least two maps varied between 15 (LG4) and 54 (LG2) (Figure 3). The information provided by each marker for the 10 genotyped parents varied from 18.5% polymorphic loci for one parent, to 21.4% for two, 15.4% for 3, 14.4% for 4, 9.1% for 5, 11% for 6 and less than 5% for 7 or more (Figure 4). The number of shared loci per linkage map decreased with the number of maps considered. In total, 89 mapped SSRs were common to two parental maps, whereas only two were common to nine parental maps.

#### Consensus maps for Q. robur and Q. petraea and comparative mapping

**Figure 5:**
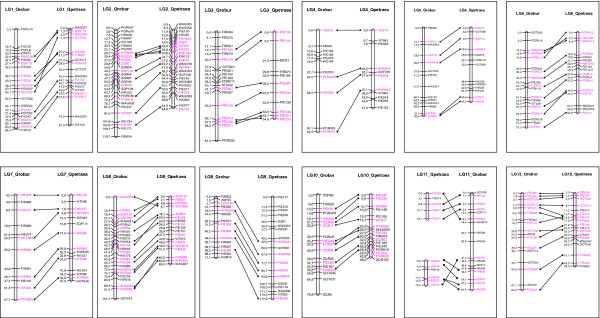
**LG consensus species maps of *Q. robur *and *Q. petraea.***

A consensus map for *Q. robur* was constructed from the seven *Q. robur* parental maps. This map includes 398 markers (including 179 accessory markers) and spans 933 cM (Table 4). Similarly, a consensus map for *Q. petraea* was established from the three parental maps available for this species. It includes 275 markers (90 accessory markers) and spans 767 cM (Table 4, Figure 5). LG sizes varied from 61.3 cM (LG11) to 116 cM (LG2) for *Q .robur* and from 31.4 cM (LG11) to 120 cM (LG9) for *Q. petraea*. Mean LG length was 78 cM for *Q. robur* and 64 cM for *Q. petraea*. The mean spacing between markers was 4.25 cM, with values ranging from 2.8 cM to 6.42 cM (Table 5).

**Table 4:** **LG size (in cM) for both species, *Q. robur *and *Q. petraea***

**n° LG**	**LG1**	**LG2**	**LG3**	**LG4**	**LG5**	**LG6**	**LG7**	**LG8**	**LG9**	**LG10**	**LG11**	**LG12**	**tot**	**mean**
*Q. robur*	84.4	116	81.5	62.3	76.8	74	63.6	101	71.1	76.6	61.3	64.2	933	77.8
*Q. petraea*	81.4	84.8	64.8	47.5	64.2	62.8	47	66.5	120	48	31.4	48.3	767	63.9

**Table 5:** **Mean distance between two loci for each LG and both species, *Q. robur *and *Q. petraea***

**mean dist loci/cM**	**LG1**	**LG2**	**LG3**	**LG4**	**LG5**	**LG6**	**LG7**	**LG8**	**LG9**	**LG10**	**LG11**	**LG12**	**mean**
*Q. robur*	2.8	1.2	2.7	3.3	2.5	2.1	2.9	2.8	2.7	2.7	2.5	2.9	2.6
*Q. petraea*	3.4	2.0	4	2.4	2.8	2.2	3.9	2.1	4.1	2.4	2.8	2.7	2.9

The consensus species maps were compared for the analysis of genomic organization and structural rearrangements. A high degree of macrocollinearity was observed between the two maps, based on 100 common markers evenly distributed over the 12 LGs (Figure 5). Some order discrepancies occurred in small sections of LGs, as in LG2 and LG3, for example. Furthermore, the positions of a few markers were inconsistent over larger distances. For example, GOT009 was localized to the top of LG1 for *Q. petraea* but was found in the center of this LG in *Q. robur*. It should also be noted that LG11 was split into two parts in *Q. petraea.*

### Transferability of EST-SSRs to other members of the Fagaceae and Nothofagaceae and comparative mapping of *Quercus* and *Castanea*

#### Transferability of EST-SSRs

We assessed the transferability of 198 EST-SSR markers to *Q. ilex* and *Q. suber*, of 194 markers to *C. sativa* and *F. sylvatica*, and 126 markers to *N. pumilio* and *N. antarctica* (Additional file 10). A PCR product of the expected size was amplified in at least one of the Fagaceae or Nothofagaceae species for 91.8% (223/243) of the EST-SSRs tested. Within the Fagaceae family, transferability was greatest for the two white oaks (*Q. faginea* and *Q. pyrenaica*), with transferability rates close to 100% (a few EST-SSRs amplified products that could not be analyzed due to extra bands and/or duplicated genes). Transferability was intermediate for *Q. sube*r, *Q. ilex* and *C. sativa,* with rates of 70.7%, 69.7% and 68% respectively. The lowest transferability within the Fagaceae family was observed in *Fagus sylvatica*, with only 14.4% of transferable markers. Levels of transferability to *Nothofagaceae* species were very low. Only 12 and 15 markers were successfully transferred to *N. pumilio* and *N. antarctica*, respectively.

#### Comparative mapping

**Figure 6:**
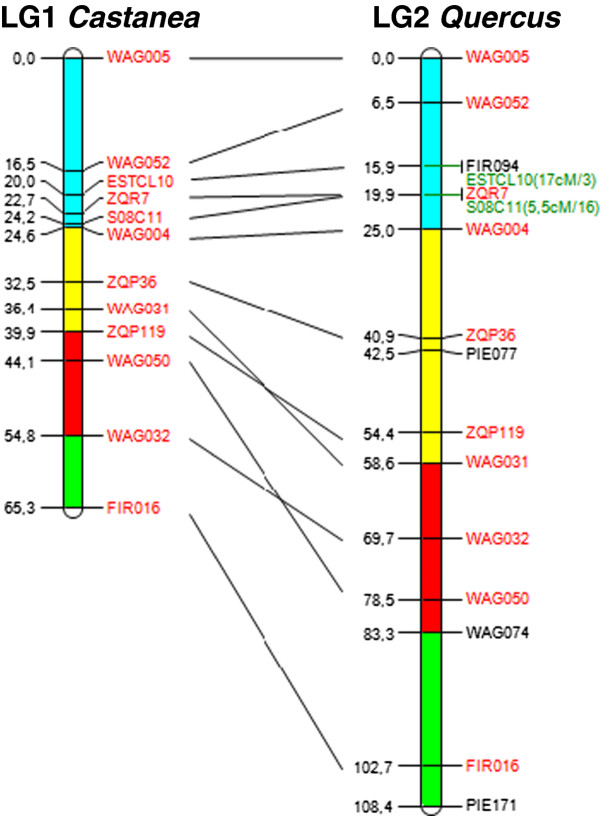
**Synteny between *Quercus *and *Castanea *for LG1.** Loci in red are common for both species, loci in green are located as accessory loci (theta/LOD), parts of linkage group which are represented by the same colour correspond to homologous segments between the two species.

We mapped 555 polymorphic markers in *Castanea* (Table 6), 91 of which were common (63 EST-SSRs, 16 gSSRs, 12 EST-P) to the consensus *Quercus* map. For all 12 *Castanea* LGs (LG-C), homologous linkage groups were identified in *Quercus* (LG-Q), with four to 17 markers shared for LG4-C (= LG5-Q) and LG1-C (= LG2-Q), respectively (Table 7). A set of 16 markers was located on linkage groups that were not homologous between *Castanea* and *Quercus*. Overall, macrosynteny was well conserved between the two genera, despite the inversion of a few markers (illustrated for one LG in Figure 6 and supported for all LGs in Additional file 11).

**Table 6:** **Segregating and mapped markers in *Castanea sativa ***

		**Castanea**	
genotyped samples		90	
polymorphic loci		555	
segregation type	1:1	502	
	1:1:1:1	50	
	1:2:1	3	
		female	male
markers type	RAPD	183	149
	AFLP	31	14
	EST-P	32	25
	gSSR	27	28
	EST-SSR	60	55
	total	333	271
distorted loci		12 (3.6%)	8 (3%)
discarded loci		20	10
unlinked loci		7	15
mapped loci		306 (92%)	246 (91%)

**Table 7:** **Correspondence between LG in *Quercus *(this study) and *Castanea *(following the nomenclature of Casasoli et al. [**[27]**])**

**LG_Q**	**LG_C**
1	6
2	1
3	8
4	2
5	4
6	11
7	5
8	7
9	9
10	10
11	3
12	12

#### Diversity analysis

A high-quality amplification product was obtained for 83.8% (166) of the 198 markers studied in the first experiment and 94.6% (157) were found to be polymorphic in at least one natural population of *Q. robur* and *Q. petraea*. In the two populations considered, expected heterozygosity (*H*_*e*_) ranged from low (0.100 and 0.091, for *Q. robur and Q. petraea*, respectively) to high (0.939 and 0.964, respectively) values. Diversity levels (allelic richness and *H*_*e*_) were similar in the two species.

Diversity levels were rather similar in the two sub-mediterranean *Quercus* species (Additional file 12). All diversity estimates were largest for EST-SSRs with dinucleotide repeat motifs. However, differences were small between tri and hexanucleotide EST-SSRs in these two oaks. The same trend was observed for *Q. robur* and *Q. petraea*.

### Oak genome evolution

#### Construction of a gene-based consensus linkage map

Individual maps were constructed from EST-SSRs (see above) and SNPs. For SNP-based markers, 105 (set 1) and 7 (set 2) candidate genes were genotyped in three mapping populations: 56 (set 1) and 4 (set 2) were localized on at least one of the six parental maps (Additional file 1 and Additional file 13). The consensus map included 381 loci (321 EST-SSRs and 60 SNP-based markers), 19 of which (18 EST-SSRs and 1 SNP) were considered to be paralogous and were assigned to different bins (on different LGs) on different individual maps. The mean number of markers mapped per LG was 32, with a maximum of 72 markers mapped for LG2 and a minimum of 22 for LG7, LG10 and LG11. Markers were assigned to 86-10 cM bins within each of the 12 linkage groups of the consensus map, for the identification of regions orthologous to regions from *Arabidopsis*, grape, poplar, *Medicago*, and soybean.

#### Synteny and duplication analysis

Independent intraspecific (*i.e.* paralogs) and interspecific (*i.e.* orthologs) comparisons are required for the precise inference of paralogous or orthologous gene relationships between oak and other eudicots and to determine the precise history of oak evolution from the known ancestor of eudicot genomes.

**Figure 7:**
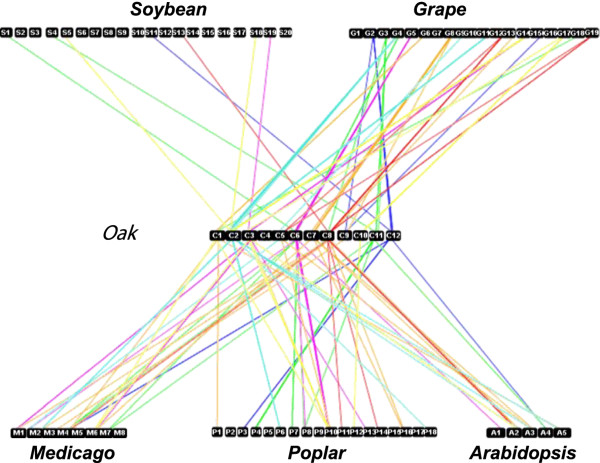
**Syntenic relationships between oak and *Arabidopsis *, grape, poplar, *Medicago, *soybean genomes.** Schematic representation of the orthologs identified between the grape chromosomes (g1 to g12) used as a reference, and the *Arabidopsis* (a1 to a5), poplar (p1 to p19), *Medicago* (m1 to m8), soybean (s1 to s20) and oak (o1 to o12) chromosomes. Each line represents an orthologous gene. The seven different colors used to represent the blocks reflect the eudicot origin from the seven ancestral chromosomes.

Using the alignment parameters and statistical tests described in the methods section, we analyzed the syntenic relationships between oak, *Arabidopsis*, poplar, *Medicago*, grape and soybean [62]. Using grape as the reference genome — this species being the closest relative of the eudicot ancestor, with a genome structured into seven protochromosomes (color code used for chromosome painting) —124 orthologous relationships were identified (Figure 7) covering 50% of the oak genome [3]. The following chromosome-to-chromosome relationships were then established (**o** for oak and **g** for grape): o1/g17, o2/g4-g11-g14, o3/g14, o5/g12-g19, o6/g4-g5, o7/g8, o8/g6-g10-g12-g19, o10/g17, o11/g3, o12/g2-g15.

**Figure 8:**
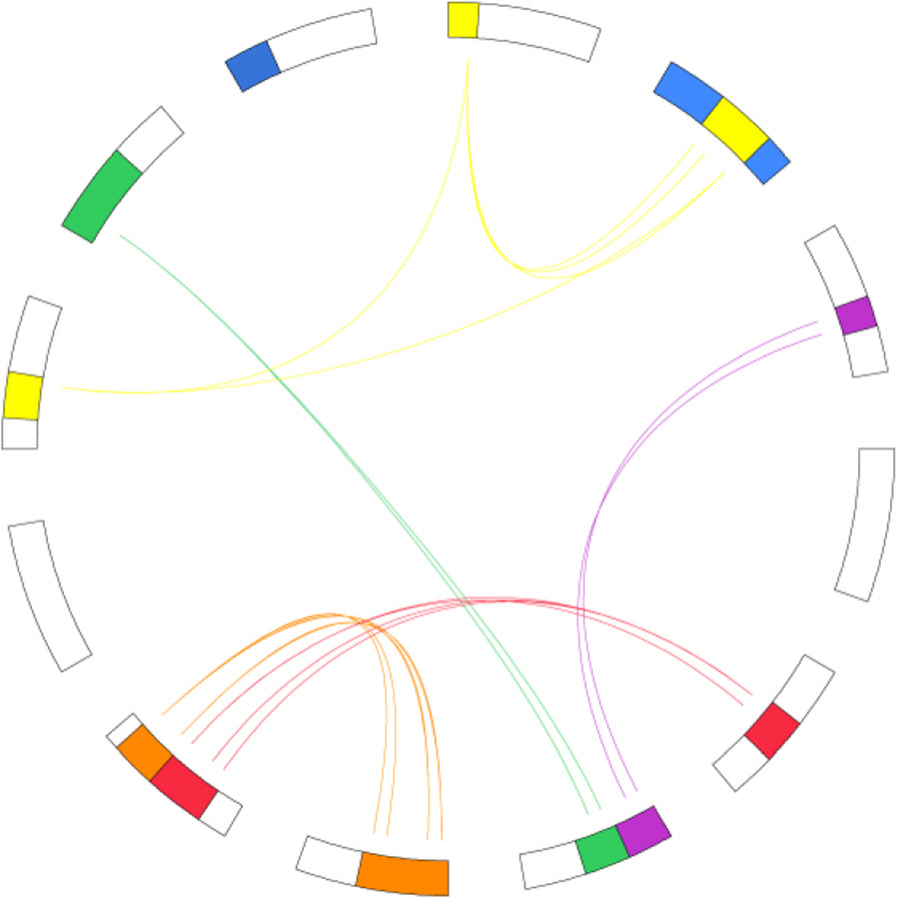
**Duplication relationships within the oak genome.** The 5 major interchromosomal duplications in oak are illustrated. Each line represents a duplicated gene. The different colours reflect the origin of the eudicots from the seven ancestral chromosomes. Duplications were visualized using the circos software (http://mkweb.bcgsc.ca/circos/.

Five major duplications were also identified, covering 28% of the genome and involving the following chromosome-to-chromosome relationships: o1-o2-o10 (yellow), o6-o11 (green), o3-o6 (purple), o7-o8 (brown), o5-o8 (red) (Figure 8).

**Figure 9:**
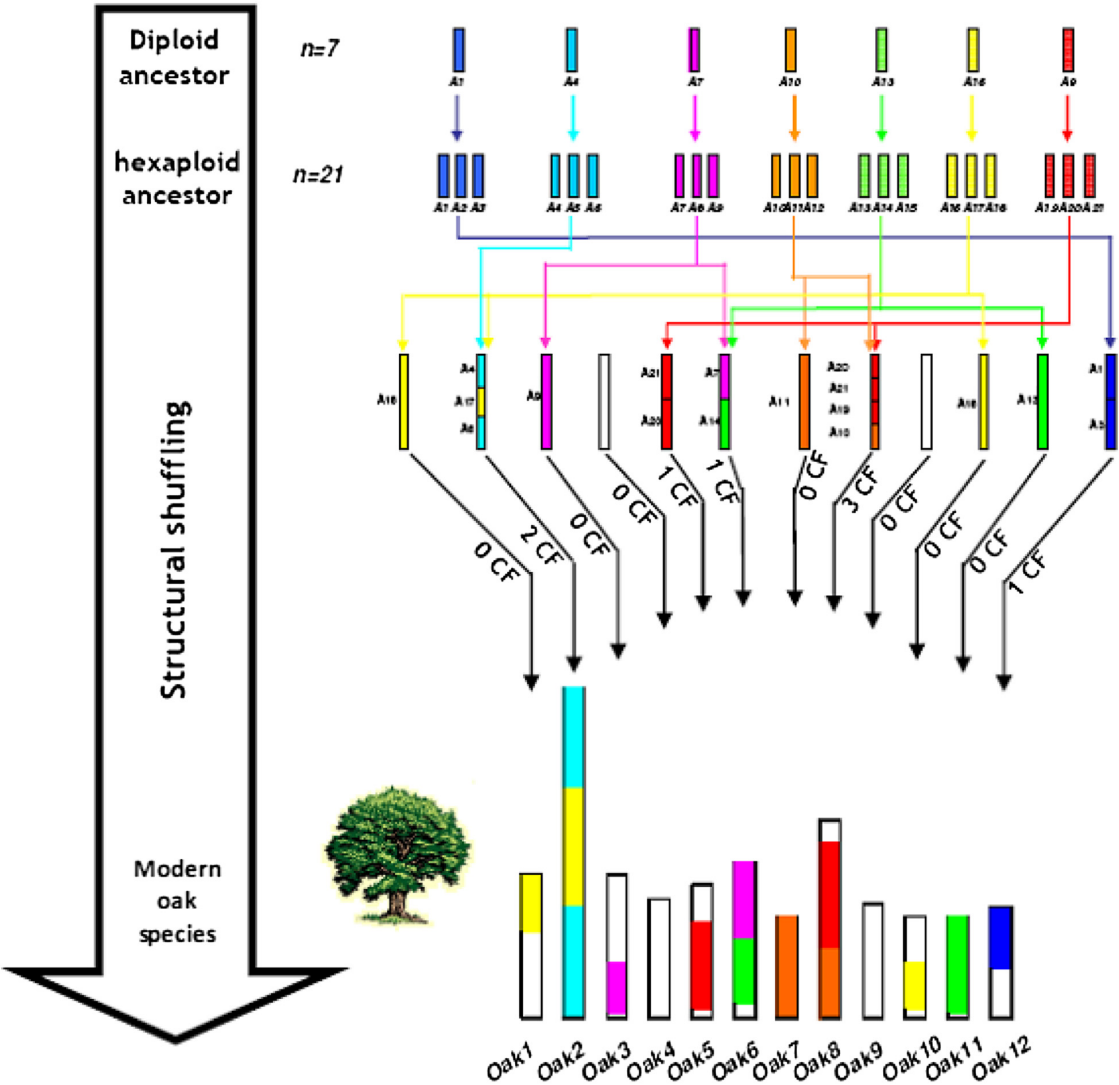
**Oak genome paleohistory.** The oak chromosomes are represented with a seven colour code to illuminate the evolution of segments from a common ancestor with seven chromosomes (A1-A7). The lineage specific shuffling events (such as chromosome fusion, CF) that have shaped the modern oak karyotype from the n = 7 or 21 ancestors are mentioned on the figure.

The integration of independent analyses of duplications within and synteny between the five major eudicot genomes led to the precise characterization in oak of five of the seven paleoduplications recently identified as the basis of the definition of seven ancestral chromosomal groups in eudicots [63]. These ancestral shared duplications were found on the following chromosome-pair combinations in oak, the locations of the seven ancestral paleoduplications in grape also being indicated : g1-g14-g17/o1-o2-o10, g2-g15-g12-g16/[not identified in oak], g3-g4-g7-g18/o6-o11, g4-g9-g11/[is partially fused into o2], g5-g7-g14/o3-o6, g6-g8-g13/o7-o8, g10-g12-g19/o5-o8. Thus, five of the seven previously identified ancestral shared duplications are characterized here for the first time in oak. Based on the ancestral and lineage-specific duplications already reported for eudicots, an evolutionary scenario can be developed in which the 12 oak chromosomes evolve from the seven chromosomes of the eudicot ancestor or, more precisely, from the 21 chromosomes resulting from polyploidization of the paleohexaploid intermediate (Figure 9). We suggest that at least eight major ancestral chromosome fusions (Cf) occurred to yield the current 12-chromosome structure, and that this process involved an intermediate ancestor that also had 12 chromosomes (Figure 9).

## Discussion

Our results provide new biological information about certain features of oak EST-SSRs, the benefits of linkage mapping with multiple pedigrees, the macrosynteny between two interfertile oak species (*Q. robur* and *Q. petraea*) and between two closely related genera (*Quercus* and *Castanea*), and about the evolution of the oak genome from the ancestor of the eudicot genome.

### Characteristics of oak EST-SSRs

As reported in other species [64], [9], dinucleotide-SSRs (di-SSRs) occurred preferentially within UTR regions, whereas trinucleotide-SSRs (tri-SSRs) that do not interfere with the reading frame occurred mostly in the coding regions of oak ESTs. The rate of polymorphism was also higher for di-SSR loci than for tri-SSR loci (72% vs. 65% (Table 3)), suggesting that SSRs occurring within UTRs are more polymorphic than those in coding regions. The number of alleles was also larger for di-SSR loci than for tri-SSR loci (60% of di-SSRs presented three or four alleles per locus, versus 37% of tri-SSRs (Table 3). A similar pattern has been reported for other species, such as castor bean [65] and cotton [66].

EST-SSRs were highly transferable between Fagaceae species, consistent with findings for other dicots, such as *Prunus*[13], *Camellia*[67], *Citrus*[15] and other species (reviewed in [10]), demonstrating a higher degree of transferability across taxonomic boundaries for EST-SSR markers than for genomic SSRs [68]. As expected, the transferability of *Quercus* EST-SSRs decreased with increasing phylogenetic distance between the species concerned. Furthermore, more than 75% of EST-SSR markers displayed high levels of genetic diversity in natural populations of *Q. robur* and *Q. petraea*. Thus, EST-SSR loci can generate sufficient polymorphism to constitute a valuable source of functional SSR markers for population genetic studies within the Fagaceae. As a proof of concept, we used two other *Quercus* species (*Q. faginea* and *Q. pyrenaica*) to provide the foundations for the use of a set of EST-SSR markers for comparative population genetic studies of the almost 20 species of deciduous oaks in the Mediterranean region. The high transferability rates into such species and the elevated polymorphims grant the use of our set of EST-SSRs for such purposes.

### Linkage mapping with multiple pedigrees

Mapping based on multiple segregating populations has several advantages over mapping based on a single pedigree. First, such strategies make it possible to map much larger numbers of markers. In this study, 274 loci were mapped in the most polymorphic mapping population (P1), but the analysis of four more pedigrees made it possible to map another 145 loci. The L-shaped distribution of the number of markers common to the different populations (Figure 4) clearly demonstrates that the number of polymorphic markers suitable for mapping increases with the number of pedigrees considered. A consensus map for oak is currently being constructed on a much larger scale, with SNP-based markers genotyped in four oak pedigrees with a total of 1,100 offspring. The addition of several thousand gene-based markers will provide a valuable tool for the alignment of genomic scaffolds from the oak genome (which is currently being sequenced) with a linkage map, with a view to establishing pseudochromosomes.

Second, based on comparisons of the positions of the mapped markers in the various populations, we identified 26 loci (6%) with different linkage group positions in different populations (25 assigned to two LGs and one assigned to three LGs), suggesting that different paralogs were indeed amplified in different genetic backgrounds, probably due to nucleotide variability at priming sites. In most cases, the discrepancies observed concerned parental maps for different pedigrees, but no such trend was identified concerning the species origin of the paralogous loci. Interestingly, eight of the 16 annotated sequences corresponding to proteins of known function belong to multiple gene families (e.g. ribosomal, RNA-binding, thioredoxin, O-methyl transferase proteins).

Finally, the establishment of linkage maps for multiple pedigrees within a species is also a prerequisite for multiple pedigree-based QTL detection strategies aiming to identify and validate QTLs in a broad genetic background [69-73]. To this end, a total of 100 EST-SSRs evenly spaced and common to the six parental maps of P1, P2 and P3 have been chosen and will be genotyped in 150 to 300 F1s for the identification of QTLs for adaptive traits (e.g. bud phenology - unpublished results).

### Comparative mapping of oak species that hybridize naturally: *Q. robur* and *Q. petraea,* and beyond

We present here the first genetic maps for two interfertile white oak species, making it possible to trace chromosomal changes [74]. A relatively large proportion (323/397; 81%) of the loci mapped was common to at least two parental maps. The integrated species maps of 397 loci covered all 12 LGs, with a mean distance between markers of 2.60 cM for *Q. robur* and 2.91 cM for *Q. petraea.* As expected, the total length of the integrated maps was greater than the length of the individual maps, as previously reported for *Vitis*[75], *Lactuca*[72] and *Picea*[76]. These results suggest that integrated maps probably cover regions not covered by the individual maps, in distal positions on the chromosomes. The genome lengths of the two consensus maps were very different — 933 cM for *Q. robur* and 767 cM for *Q. petraea —* despite the similar physical size of the two genomes [77]. This discrepancy may reflect differences in recombination rates between *Q. robur* and *Q. petraea* or differences in recombination rate in these particular genotypes. The overall macrocollinearity between these two species maps was high, with little shuffling of marker order between homologous LGs. Some local inconsistencies in marker order were observed, as reported for other species [72,75,78,79], but no duplication or major chromosomal rearrangement (inversion, translocation) was characterized. This high degree of collinearity should facilitate the identification of genomic islands involved in species differentiation [80,81].

A comparison of the consensus maps of *Quercus* and *Castanea* revealed a high degree of collinearity and synteny between the 12 homologous linkage groups, despite the divergence of their lineages 70 million years ago [82]. A search for genes underlying similar QTLs, based on comparative mapping, could be considered, making use of the sequencing data available for *Castanea*.

### Oak genome evolution

We have identified precise chromosomal relationships within the oak genome corresponding to the ancestral hexaploidization event reported in eudicots [83]. This made it possible to propose an evolutionary scenario describing the development of the modern oak genome from the ancestral eudicot karyotype over the last 100 million years. Such information is of prime importance for gene cloning and, for example, detecting gene function by complementing *Arabidopsis* mutants. The ancestral hexaploidization event in eudicots generated two additional copies for any ancestral gene function considered [3]. In modern eudicot species, these three homologous copies may have or may not have retained the same function as the ancestral gene. It is thus of the utmost importance, when cloning candidate genes on the basis of synteny (*i.e.* translational genomics approach with the use of reference (*i.e.* sequenced) genomes), to investigate all the duplicated copies, which may prove to be redundant or complementary in terms of their function and the phenotype they confer [84].

## Conclusion

This study provides new insights into the distribution of EST-derived SSRs between five mapping populations of two oak species and the benefits of using multiple pedigrees for the construction of consensus maps. We mapped 397 loci, 81% of which were common to at least two different mapping populations. The level of conserved macrosynteny was very high between *Q. robur* and *Q. petraea*, as well as between *Quercus* spp. and *Castanea sativa*, opening perspectives for QTL validation across phylogenetically related species as demonstrated by Faivre Rampant *et al*. [85].

Functional characterization of these EST-derived oak SSRs revealed many genes with biological, cellular and molecular functions. Their position is now being compared to that of already mapped QTLs and suggest putative positional candidate genes that are being used as anchor markers to fine map large effect QTLs (e.g. for water use efficiency and bud burst) and identify the underlying sub genomic region using the BAC libraries available for *Quercus robur*[85].

## Authors' contributions

CB1, EC, OG, JD, HPK, PGG: developing and genotyping EST-SSRs in oak mapping populations; FB, GGV, CS, FV, CM: EST-SSR transferability in the Fagaceae and Nothofagaceae, diversity analysis in natural populations of oaks, and contribution to the genotyping of oak and chestnut pedigrees; PGG: transferability and polymorphism analysis of EST-SSRs in the sub-mediterranean oaks; CB1, EC, CP: linkage mapping; EC, FA, CB2: SNP analysis; FM, JS, CP analysis of oak genome evolution; FV, GR: plant material preparation; JPS: database entries and analysis, CB1, CP, JS, manuscript preparation, CB1, CP, AK: project design; CP, AK: funding and overall supervision. All the authors have read and approved the manuscript.

## Supplementary Material

Additional file 1 Description of SSR markers (locus name, forward and reverse primers, EST sequences, accessions number). Click here for file

Additional file 2 **Description of location of the *Q. robur *and *Q. petraea *individuals used for diversity analyses.** Click here for file

Additional file 3 **Map of the populations location of *Q. faginea *and *Q; pyrenaica *used for the diversity analyses.** Click here for file

Additional file 4 Description of the SSCP method implemented on a Licor DNA sequencer. Click here for file

Additional file 5 **Comparison of Gene Ontology classification between the four sets of sequences containing EST-SSRs (CR: Coding Region, NCR: Non Coding Region, 5’UTR: 5’ Un-Transcribed Region, 3’UTR: 3’ Un-Transcribed Region.** The relative frequencies of GO hits for oak sequences are assigned to the GO functional categories (Cellular Compounds, Molecular Function and Biological Process. Click here for file

Additional file 6 Clustering of Blast2GO categories using the four sets of sequences containing EST (CR: Coding Region, NCR: Non Coding Region, 5’UTR: 5’ Un-Transcribed Region, 3’UTR: 3’ Un-Transcribed Region). Click here for file

Additional file 7 Number of loci per LG for the five pedigrees (in () data for two partial linkage group, acc: accessory markers, tot: total number of markers). Click here for file

Additional file 8 Map length in cM of the ten parental maps (in () are written the values obtained for two partial linkage groups). Click here for file

Additional file 9 Estimation of genome length in cM for LOD score ranging from 3 to 5. Click here for file

Additional file 10 **Transferability results for EST-SSR loci tested on *Castanea sativa, Fagus sylvatica, Quercus faginea, Q. pyrenaica, Q. ilex, Q. suber, Nothofagus pumilio *and *N. antartica***. Click here for file

Additional file 11 **Linkage groups homology between *Castanea *and *Quercus***. Click here for file

Additional file 12 **Diversity results for *Quercus robur ***, ***Q. petraea; Q. faginea *and *Q. pyrenaica *(LG: Linkage group, AR: allelic richness, H**_**o**_**: observed heterozygosity, H**_**e**_**: expected heterozygosity).** Click here for file

Additional file 13 List of the all mapped SNPs, annotation and map location. Click here for file

## References

[B1] Bernardo R Molecular markers and selection for complex traits in plants: Learning from the last 20 years Crop Science 2008 48 5 1649 1664 10.2135/cropsci2008.03.0131

[B2] Bergelson J Roux F Towards identifying genes underlying ecologically relevant traits in *Arabidopsis thaliana* Nat Rev Genet 2010 11 867 879 10.1038/nrg2896 21085205

[B3] Abrouk M Murat F Pont C Messing J Jackson S Faraut T Tannier E Plomion C Cooke R Feuillet C Salse J Paleogenomics of plants: modern species synteny-based modelling of extinct ancestors Trends Plant Sci 2010 15 479 487 10.1016/j.tplants.2010.06.001 20638891

[B4] Troggio M Malacarne G Coppola G Segala C Dustin A Cartwright DA Pindo M Stefanini M Mank R Moroldo M Morgante M Grando MS Velasco R A Dense Single-Nucleotide Polymorphism-Based Genetic Linkage Map of Grapevine (*Vitis vinifera* L.) Anchoring Pinot NoirBacterial Artificial Chromosome Contigs Genetics 2007 176 2637 2650 10.1534/genetics.106.067462 17603124 PMC1950661

[B5] Tanksley SD Martin W Ganal MW Martin GD Chromosome landing: a paradigm for map-based gene cloning in plants with large genomes Trends Genet 1995 11 63 68 10.1016/S0168-9525(00)88999-4 7716809

[B6] Remington DL Ungerer MC Purugganan MD Map-based cloning of quantitative trait loci: progress and prospects Genet Res 2001 78 213 218 10.1017/S0016672301005456 11865710

[B7] Lefebvre-Pautigny F Wu F Philippot M Rigoreau M Priyono Zouine M Frasse P Bouzayen M Broun P Pétiard V Tanskley SD Crouzillat D High resolution synteny maps allowing direct comparisons between the coffee and tomato genomes Tree Genetics Genomes 2010 6 4 565 577 10.1007/s11295-010-0272-3

[B8] Muñoz A Zheng C Zhu Q Albert VA Rounsley S Sankoff D Scaffold filling, contig fusion and comparative gene order inference BMC Bioinformatics 2010 11 304 10.1186/1471-2105-11-304 20525342 PMC2902449

[B9] Studer B Asp T Frei U Hentrup S Meally H Guillard A Barth S Muylle H Roldán-Ruiz I Barre P Expressed sequence tag-derived microsatellite markers of perennial ryegrass (*Lolium perenne* L.) Mol Breeding 2008 21 533 548 10.1007/s11032-007-9148-0

[B10] Ellis JR Burke JM EST-SSR as a resource for population genetic analyses Heredity 2007 99 125 132 10.1038/sj.hdy.6801001 17519965

[B11] Fouet O Allegre M Argout X Jeanneau M Lemainque A Pavek S Boland A Risterucci AM Loor G Tahi M Sabau X Courtois B Lanaud C Structural characterization and mapping of functional EST-SSR markers in *Theobroma cacao* Tree Genetics and Genomes 2011 7 799 817 10.1007/s11295-011-0375-5

[B12] Moccia M Oger-Desfeux C Marais G Widmer AA White Campion (*Silene latifolia*) floral expressed sequence tag (EST) library: annotation, EST-SSR characterization, transferability, and utility for comparative mapping BMC Genomics 2009 10 243 257 10.1186/1471-2164-10-243 19467153 PMC2689282

[B13] Mnejja M Garcia-Mas J Audergon JM Arús P Prunus microsatellite marker transferability across rosaceous crops Tree Genetics and Genomes 2010 10 689 700

[B14] Xie WG Zhang XQ Cai HW Liu W Peng Y Genetic diversity analysis and transferability of cereal EST-SSR markers to orchardgrass (*Dactylis glomerata* L.) Biochem Syst Ecol 2010 38 4 740 749 10.1016/j.bse.2010.06.009

[B15] Luro F Costantino G Terol J Argout X Allario T Wincker P Talon M Ollitrault P Morillon R Transferability of the EST-SSRs developed on Nules clementine (*Citrus clementina* Hort ex Tan) to other Citrus species and their effectiveness for genetic mapping BMC Genomics 2008 9 287 300 10.1186/1471-2164-9-287 18558001 PMC2435559

[B16] Kremer A Vinceti B Alia R Burczyk J Cavers S Degen D Finkeldey R Fluch S Gömöry D Gugerli F Koelewijn HP Koskela J Lefèvre F Morgante M Mueller-Starck G Plomion C Taylor G Turok J Savolainen O Ziegenhagen B Forest Ecosystem genomics and adaptation: Evoltree Conference Report Trees Genetics and Genomes 2011 7 869 875 10.1007/s11295-011-0378-2

[B17] Barreneche T Bodénès C Lexer C Trontin JF Fluch S Streiff R Plomion C Roussel G Steinkellner H Burg K A genetic linkage map of *Quercus robur* L. (pedunculate oak) based on RAPD, SCAR, microsatellite, minisatellite, isozyme and 55 rDNA markers Theor Appl Genet 1998 97 7 1090 1103 10.1007/s001220050996

[B18] Casasoli M Mattioni C Cherubini M Villani F A Genetic Linkage map of European chestnut (*Castanea sativa* Mill).based on RAPD, ISSR and isozyme markers. Theor Appl Genet 2001 102 8 1190 1199 10.1007/s00122-001-0553-1

[B19] Kubisiak TL Hebard FV Nelson CD Zhang J Bernatzky R Huang H Anagnostakis SL Doudrick RL Molecular mapping of resistance to blight in an interspecific cross in the genus *castanea* Phytopathology 1997 87 7 751 759 10.1094/PHYTO.1997.87.7.751 18945098

[B20] Scalfi M Troggio M Piovani P Leonardi S Magnaschi G Vendramin GG Menozzi P A RAPD, AFLP and SSR linkage map, and QTL analysis in European beech(*Fagus sylvatica* L.). Theor Appl Genet 2004 108 3 433 441 10.1007/s00122-003-1461-3 14574454

[B21] Scotti-Saintagne C Bodénès C Barreneche T Bertocchi E Plomion C Kremer A Detection of quantitative trait loci controlling bud burst and height growth in *Quercus robur* L Theor Appl Genet 2004 109 8 1648 1659 10.1007/s00122-004-1789-3 15490107

[B22] Derory J Scotti-Saintagne C Bertocchi E Le Dantec L Graignic N Jauffres A Casasoli M Chancerel E Bodénès C Alberto F Kremer A Contrasting correlations between diversity of candidate genes and variation of bud burst in natural and segregating populations of European oaks Heredity 2010 104 438 448 10.1038/hdy.2009.134 19812610

[B23] Gailing O Langenfeld-Heyser R Polle A Finkeldey R QTL loci affecting stomatal density and growth in a *Quercus robur* progeny: implications for the adaptation to changing environments Glob Chang Biol 2008 14 1934 1946 10.1111/j.1365-2486.2008.01621.x

[B24] Parelle J Zapater M Scotti-Saintagne C Kremer A Jolivet Y Dreyer E Brendel O Quantitative trait loci of tolerance to waterlogging in a European oak (*Quercus robur* L.): physiological relevance and temporal effect patterns Plant Cell Environ 2007 30 4 422 434 10.1111/j.1365-3040.2006.01629.x 17324229

[B25] Brendel O Le Thiec D Scotti-Saintagne C Bodénès C Kremer A Guehl JM Quantitative trait loci controlling water use efficiency and related traits in *Quercus robur* L Tree Genet Genomes 2008 4 2 263 278 10.1007/s11295-007-0107-z

[B26] Barreneche T Casasoli M Russell K Akkak A Meddour H Plomion C Villani F Kremer A Comparative mapping between *Quercus* and *Castanea* using simple-sequence repeats (SSRs) Theor Appl Genet 2004 108 3 558 566 10.1007/s00122-003-1462-2 14564395

[B27] Casasoli M Derory J Morera-Dutrey C Brendel O Porth I Guehl JM Villani F Kremer A Comparison of Quantitative Trait Loci for Adaptive Traits Between Oak and Chestnut Based on an Expressed Sequence Tag Consensus Map Genetics 2006 172 533 546 16204213 10.1534/genetics.105.048439 PMC1456181

[B28] Casasoli M Pot D Plomion C Monteverdi MC Barreneche T Lauteri M Villani F Identification of QTLs affecting adaptive traits in *Castanea sativa* Mill Plant Cell Env 2004 27 9 1088 1101 10.1111/j.1365-3040.2004.01214.x

[B29] Durand J Bodénès C Chancerel E Frigerio JM Vendramin G Sebastiani F Buonamici A Gailing O Koelewijn HP Villani F Mattioni C Cherubini M Goicoechea PG Herrán A Ikaran Z Cabané C Ueno S Alberto F Dumoulin PY Guichoux E de Daruvar A Kremer A Plomion C A fast and cost-effective approach to develop and map EST-SSR markers: oak as a case study BMC Genomics 2010 11 570 583 10.1186/1471-2164-11-570 20950475 PMC3091719

[B30] Grattapaglia D Sederoff R Genetic linkage maps of *Eucalyptus grandis* and *Eucalyptus urophylla* using a pseudo-testcross: mapping strategy and RAPD markers Genetics 1994 137 4 1121 1137 7982566 10.1093/genetics/137.4.1121 PMC1206059

[B31] Berlin S Lagercrantz U von Arnold S Öst T Rönnberg-Wästljung AC High-density linkage mapping and evolution of paralogs and orthologs in *Salix* and *Populus* BMC Genomics 2010 11 129 143 10.1186/1471-2164-11-129 20178595 PMC2834636

[B32] McConnell M Mamidi S Lee R Chikara S Rossi M Papa R McClean P Syntenic relationships among legumes revealed using a gene-based genetic linkage map of common bean (*Phaseolus vulgaris* L.) Theor Appl Genet 2010 121 6 1103 1116 10.1007/s00122-010-1375-9 20607211

[B33] Ueno S Le Provost G Léger V Klopp C Noirot C Frigerio J-M Salin F Salse J Abrouk M Murat F Brendel O Derory J Abadie P Leger P Cabane C Barré A de Daruvar A Couloux A Wincker P Reviron M-P Kremer A Plomion C Bioinformatic analysis of ESTs collected by Sanger and pyrosequencing methods for a keystone forest tree species: oak BMC Genomics 2010 11 650 674 10.1186/1471-2164-11-650 21092232 PMC3017864

[B34] The Gene Ontology Consortium Gene ontology: tool for the unification of biology Nat Genet 2000 25 1 x25 29 10.1038/75556 PMC3037419 10802651

[B35] Conesa A Götz S García-Gómez JM Terol J Talón M Robles M Blast2GO: a universal tool for annotation, visualization and analysis in functional genomics research Bioinformatics 2005 15 21(18) 3674 3680 16081474 10.1093/bioinformatics/bti610

[B36] Shamir R Maron-Katz A Tanay A Linhart C Steinfeld I Sharan R Shiloh Y Elkon R EXPANDER an integrative program suite for microarray data analysis BMC Bioinformatics 2005 6 232 244 10.1186/1471-2105-6-232 16176576 PMC1261157

[B37] Dow BD Ashley MV Howe HF Characterization of highly variable (GA/CT)n microsatellites in the bur oak, *Quercus macrocarpa* Theor Appl Genet 1995 91 137 141 10.1007/BF00220870 24169679

[B38] Isagi Y Suhandono S PCR primers amplifying microsatellite loci of *Quercus myrsinifolia* Blume and their conservation between oak species Mol Ecol 1997 6 897 899 10.1111/j.1365-294X.1997.tb00147.x 9301079

[B39] Steinkellner H Fluch S Turetschek E Lexer C Streiff R Kremer A Burg K Glössl J Identification and characterization of (GA/CT)n- microsatellite loci from *Quercus petraea* Plant Mol Biol 1997 33 6 1093 1096 10.1023/A:1005736722794 9154990

[B40] Kampfer S Lexer C Glössl J Steinkellner H Characterization of (GA)n microsatellite loci from *Quercus robur* Hereditas 1998 129 183 186

[B41] Lexer C Heinze B Steinkellner H Kampfer S Ziegenhagen B Glosssl J Microsatellite analysis of maternal half-sib families of *Quercus robur*, pedunculate oak: detection of seed contaminations and inference of the seed parents from the offspring Theor Appl Genet 1999 99 185 191 10.1007/s001220051223

[B42] Aldrich PR Michler CH Weilin S Romero-Severson J Microsatellite markers for northern red oak (Fagaceae: *Quercus rubra*) Molecular Ecology Notes 2002 2 4 472 474 10.1046/j.1471-8286.2002.00282.x

[B43] Buck EJ Hadonou M James CJ Blakesley D Russell K Isolation and characterization of polymorphic microsatellites in European chestnut (*Castanea sativa* Mill.) Mol. Ecol. Notes 2003 3 239 241 10.1046/j.1471-8286.2003.00410.x

[B44] Marinoni D Akkak A Bounous G Edwards KJ Botta R Development and characterization of microsatellite markers in *Castanea sativa* (Mill.) Mol. Breed 2003 11 127 136 10.1023/A:1022456013692

[B45] Schuelke M An economic method for the fluorescent labeling of PCR fragments Nat Biotechnol 2000 18 2 233 234 10.1038/72708 10657137

[B46] Van Ooijen JW Kyazma BV JoinMap® 4 Software for the calculation of genetic linkage maps in experimental populations 2006 Netherlands: Wageningen

[B47] Voorrips RE MapChart: Software for the graphical presentation of linkage maps and QTLs J Hered 2002 93 1 77 78 10.1093/jhered/93.1.77 12011185

[B48] Hulbert SH Ilott TW Legg EJ Lincoln SE Lander ES Michelmore RW Genetic analysis of the fungus, *Bremia lactucae*, using restriction fragment length polymorphisms Genetics 1988 120 947 958 2906309 10.1093/genetics/120.4.947 PMC1203586

[B49] Chakravarti A Lasher L Reefer JE A maximum likehood method for estimating genome length using genetic linkage data Genetics 1991 128 175 182 2060775 10.1093/genetics/128.1.175 PMC1204446

[B50] Goudet J FSTAT, a program to estimate and test gene diversities and fixation indices (version 2.9.3) 2001 Available from http://www.unil.ch/izea/softwares/fstat.html. Updated from Goudet (1995).

[B51] Alberto F Niort J Derory J Lepais O Vitalis R Galop D Kremer A Population differentiation of sessile oak at the altitudinal front of migration in the French Pyrenees Mol Ecol 2010 19 13 2626 2639 10.1111/j.1365-294X.2010.04631.x 20561196

[B52] Derory J Leger P Garcia V Schaeffer J Hauser MT Salin F Luschnig C Plomion C Glossl J Kremer A Transcriptome analysis of bud burst in sessile oak (*Quercus petraea*) New Phytol 2006 170 4 723 738 10.1111/j.1469-8137.2006.01721.x 16684234

[B53] Lepoittevin C Frigerio JM Garnier-Géré P Salin F Cervera MT Vornam B Harvengt L Plomion C In vitro vs in silico detected SNPs for the development of a genotyping array: what can we learn from a non-model species? PLoS One 2010 5 6 e11034 10.1371/journal.pone.0011034 20543950 PMC2882948

[B54] Alberto FJ Derory J Boury C Frigerio JM Zimmermann NE Kremer A Imprints of natural selection along environmental gradients in phenology related genes of Quercus petraeasubmitted. 10.1534/genetics.113.153783 PMC3781976 23934884

[B55] Wegrzyn JL Lee JM Liechty J Neale DB PineSAP–sequence alignment and SNP identification pipeline Bioinformatics 2009 25 19 2609 2610 10.1093/bioinformatics/btp477 19667082 PMC2752621

[B56] Orita M Iwahana H Kanazawa H Hayashi K Sekiya T Detection of polymorphisms of human DNA by gel electrophoresis as single-strand conformation polymorphism Proc Natl Acad Sci USA 1989 86 2766 2770 10.1073/pnas.86.8.2766 2565038 PMC286999

[B57] Hsu TM Chen X Duan S Miller RD Kwok PY Universal SNP genotyping assay with fluorescence polarization detection Biotechniques 2001 31 560 570 11570500 10.2144/01313rr01

[B58] Plomion C O’Malley DM Recombination rate differences for pollen parents and seed parents in pine Heredity 1996 77 341 350 10.1038/hdy.1996.152

[B59] Kumar S Tamura K Nei M Mega3: integrated software for Molecular Evolutionary Genetics Analysis and sequence alignment Brief Bioinform 2004 5 150 163 10.1093/bib/5.2.150 15260895

[B60] Gaut BS Morton BR McCaig BC Clegg MT Substitution rate comparisons between grasses and palms: Synonymous rate differences at the nuclear gene *Adh* parallel rate differences at the plastid gene *rbcL* Proc Natl Acad Sci USA 1996 93 10274 10279 10.1073/pnas.93.19.10274 8816790 PMC38374

[B61] SanMiguel P Gaut BS Tikhonov A Nakajima Y Bennetzen JL The paleontology of intergene retrotransposons of maize Nat Genet 1998 20 43 45 10.1038/1695 9731528

[B62] Salse J Abrouk M Murat F Masood Quraishi U Feuillet C Improved standards and new comparative genomics tools provide new insights into grasses paleogenomics Briefings in Bioinf 2009 10 6 619 630 10.1093/bib/bbp037 19720678

[B63] Salse J Abrouk M Bolot S Guilhot N Courcelle E Faraut T Waugh R Close TJ Messing J Feuillet C Reconstruction of monocotelydoneous proto-chromosomes reveals faster evolution in plants than in animals PNAS USA 2009 106 14908 14913 10.1073/pnas.0902350106 19706486 PMC2736418

[B64] Blanca J Cañizares J Roig C Ziarsolo P Nuez F Pico B Transcriptome characterization and high throughput SSRs and SNPs discovery in *Curcubita pepo* (Cucurbitaceae) BMC Genomics 2011 12 104 119 10.1186/1471-2164-12-104 21310031 PMC3049757

[B65] Qiu L Yang C Yang JB Liu A Exploiting EST databases for the development and characterization of EST-SSR markers in castor bean (*Ricinus communis* L.) BMC Plant Biol 2010 10 278 288 10.1186/1471-2229-10-278 21162723 PMC3017068

[B66] Yu Y Daojun Yuan D Liang S Li X Wang X Lin Z Zhang X Genome structure of cotton revealed by a genome-wide SSR genetic map constructed from a BC1 population between *Gossypium hirsutum* and *G. barbadense* BMC Genomics 2011 12 15 29 10.1186/1471-2164-12-15 21214949 PMC3031231

[B67] Sharma RK Bhardwaj P Negi R Mohapatra T Ahuja PS Identification, characterization and utilization of unigene derived microsatellite markers in tea (*Camellia sinensis* L.) BMC Plant Biol 2009 9 53 77 10.1186/1471-2229-9-53 19426565 PMC2693106

[B68] Varshney RK Graner A Sorrells ME Genic microsatellite markers in plants: features and applications Trends Biotechnol 2005 23 1 48 55 10.1016/j.tibtech.2004.11.005 15629858

[B69] Danan S Veyrieras JB Lefebvre V Construction of a potato consensus map and QTL meta-analysis offer new insights into the genetic architecture of late blight resistance and plant maturity traits BMC Plant Biol 2011 11 16 10.1186/1471-2229-11-16 21247437 PMC3037844

[B70] De Keyser E Shu QY Bockstaele EV De Riek J Multipoint-likelihood maximization mapping on 4 segregating populations to achieve an integrated framework map for QTL analysis in pot azalea (*Rhododendron simsii* hybrids) BMC Mol Biol 2010 11 1 21 10.1186/1471-2199-11-1 20070894 PMC2837023

[B71] Truntzler Barrière Y Sawkins MC Lespinasse D Betran J Charcosset A Moreau L Meta-analysis of QTL involved in silage quality of maize and comparison with the position of candidate genes Theor Appl Genet 2010 121 8 1465 1482 10.1007/s00122-010-1402-x 20658277

[B72] Truco MJ Antonise R Lavelle D Ochoa O Kozik A Witsenboer H Fort SB Jeuken MJW Kesseli RV Lindhout P Michelmore RW Peleman J A high-density, integrated genetic linkage map of lettuce (*Lactuca* spp.) Theor Appl Genet 2007 115 735 746 10.1007/s00122-007-0599-9 17828385

[B73] Blanc G Charcosset A Mangin B Gallais A Moreau L Connected populations for detecting quantitative trait loci and testing for epistasis: an application in maize Theor Appl Genet 2006 113 206 224 10.1007/s00122-006-0287-1 16791688

[B74] Wu F Tanksley SD Chromosomal evolution in the plant family Solanaceae BMC Genomics 2010 11 182 193 10.1186/1471-2164-11-182 20236516 PMC2847972

[B75] Vezzulli S Troggio M Coppola G Jermakow A Cartwright D Zharkikh A Stefanini M Grando MS Viola R Adam-Blondon AF Thomas M This P Velasco R A reference integrated map for cultivated grapevine (*Vitis vinifera* L.) from three crosses, based on 283 SSR and 501 SNP-based markers Theor Appl Genet 2008 117 499 511 10.1007/s00122-008-0794-3 18504538

[B76] Pelgas B Bousquet J Beauseigle S Isabel N A composite linkage map from two crosses for the species complex *Picea mariana* × *Picea rubens* and analysis of synteny with other Pinaceae Theor Appl Genet 2005 111 1466 1488 10.1007/s00122-005-0068-2 16215729

[B77] Kremer A Casasoli M Barreneche T Bodénès C Sisco P Kubisiak T Scalfi M Leonardi S Bakker EG Buiteveld J Romero-Severson J Arumuganathan K Derory J Scotti-Saintagne C Roussel G Bertocchi ME Lexer C Porth I Hebard F Clark C Carlson J Plomion C Koelewijn H Villani F Kole CR Fagaceae trees Genome Mapping & Molecular Breeding. Forest Trees 2007 Heidelberg, New York, Tokyo: Springer-Verlag, Berlin 16 187 5.

[B78] N’Diaye A Van de Weg WE Kodde LP Koller B Dunemann F Thiermann M Tartarini S Gennari F Durel CE Construction of an integrated consensus map of the apple genome based on four mapping populations Tree Genet Genomes 2008 4 727 743 10.1007/s11295-008-0146-0

[B79] Doligez A Adam-Blondon AF Cipriani G Di Gaspero G Laucou V Merdinoglu D Meredith CP Riaz S Roux SC This P An integrated SSR map of grapevine based on five mapping populations Theor Appl Genet 2006 113 3 369 382 10.1007/s00122-006-0295-1 16799809

[B80] Saintagne C Bodénès C Barreneche T Pot D Plomion C Kremer A Distribution of genomic regions differentiating oak species assessed by QTL detection Heredity 2004 92 1 20 30 10.1038/sj.hdy.6800358 14508500

[B81] Abadie P Roussel G Dencausse B Bonnet C Bertocchi E Louvet JM Kremer A Garnier-Géré P Strength, diversity and plasticity of postmating reproductive barriers between two hybridizing oak species (*Quercus robur* L. and *Quercus petraea* (Matt) Liebl.). J Evol Biol 2011 25 157 173 22092648 10.1111/j.1420-9101.2011.02414.x

[B82] Manos PS Z-K Zhou ZK Cannon CH Systematics of Fagaceae: phylogenetic tests of reproductive trait evolution International Journal of Plant Sciences 2001 162 1361 1379 10.1086/322949

[B83] Jaillon O Jean-Marc Aury JM Noel B Policriti A Clepet C The grapevine genome sequence suggests ancestral hexaploidization in major angiosperm phyla Nature 2007 449 463 467 10.1038/nature06148 17721507

[B84] Salse J In silico archeogenomics unveils modern plant genome organisation, regulation and evolution Curr Opin Plant Biol 2012 15 1 9 10.1016/j.pbi.2011.12.001 22280839

[B85] Faivre-Rampant P Lesur I Boussardon C Bitton F Martin-Magniette ML Bodénès C Le Provost G Berges H Fluch S Kremer A Plomion C Analysis of BAC end sequences in oak, a keystone forest tree species, providing insight into the composition of its genome BMC Genomics 2011 12 292 305 10.1186/1471-2164-12-292 21645357 PMC3132169

