# Supplementary material for: Comparative mapping in the Fagaceae and beyond with EST-SSRs
Source: BMC Plant Biol. 2012 Aug 29;12:153. doi: 10.1186/1471-2229-12-153 (PMC3493355; doi:10.1186/1471-2229-12-153)
Supplement: Additional file 2 — Description of location of the Q. robur and Q. petraea individuals used for diversity analyses. [file 1471-2229-12-153-S2.docx]

Description of individuals used for *Q. robur* and *Q. petraea* diversity analyses

| species name | Population name | Country | longitude, latitude |
| --- | --- | --- | --- |
| Q. petraea | Arlaban | Spain | 42,97°N, 2,55°W |
| Q. petraea | Büren | Switzerland | 47,10°N, 7,38°E |
| Q. petraea | De Meinweg | The Netherlands | 51,18°N, 6,14°E |
| Q. petraea | Petite Charnie | France | 48,1°N, 0,15°W |
| Q. petraea | Petite Charnie | France | 48,1°N, 0,15°W |
| Q. petraea | Orleans | France | 43,83°N, 1,91°E |
| Q. petraea | Orleans | France | 43,83°N, 1,91°E |
| Q. petraea | Orleans | France | 43,83°N, 1,91°E |
| Q. petraea | Rantzau | Germany | 53,72°N, 9,77°E |
| Q. petraea | Rantzau | Germany | 53,72°N, 9,77°E |
| Q. petraea | Roudsea Wood | United Kingdom | 54,22°N, 3,02°W |
| Q. petraea | Sopron | Hungary | 47,53°N, 16,5°E |
| Q. robur | Pierroton | France | 44,44°N, 0,46°W |
| Q. robur | Pierroton | France | 44,44°N, 0,46°W |
| Q. robur | Arcachon | France | 44,30°N, 1,11°W |
| Q. robur | Arlaban | Spain | 42,97°N, 2,55°W |
| Q. robur | Büren | Switzerland | 47,10°N, 7,38°E |
| Q. robur | De Meinweg | The Netherlands | 51,18°N, 6,14°E |
| Q. robur | Petite Charnie | France | 48,1°N, 0,15°W |
| Q. robur | Rantzau | Germany | 53,72°N, 9,77°E |
| Q. robur | Rantzau | Germany | 53,72°N, 9,77°E |
| Q. robur | Roudsea Wood | United Kingdom | 54,22°N, 3,02°W |
| Q. robur | Roudsea Wood | United Kingdom | 54,22°N, 3,02°W |
| Q. robur | Sopron | Hungary | 47,53°N, 16,5°E |
