# Supplementary figures and images for: Comparative mapping in the Fagaceae and beyond with EST-SSRs
Source: BMC Plant Biol. 2012 Aug 29;12:153. doi: 10.1186/1471-2229-12-153 (PMC3493355; doi:10.1186/1471-2229-12-153)

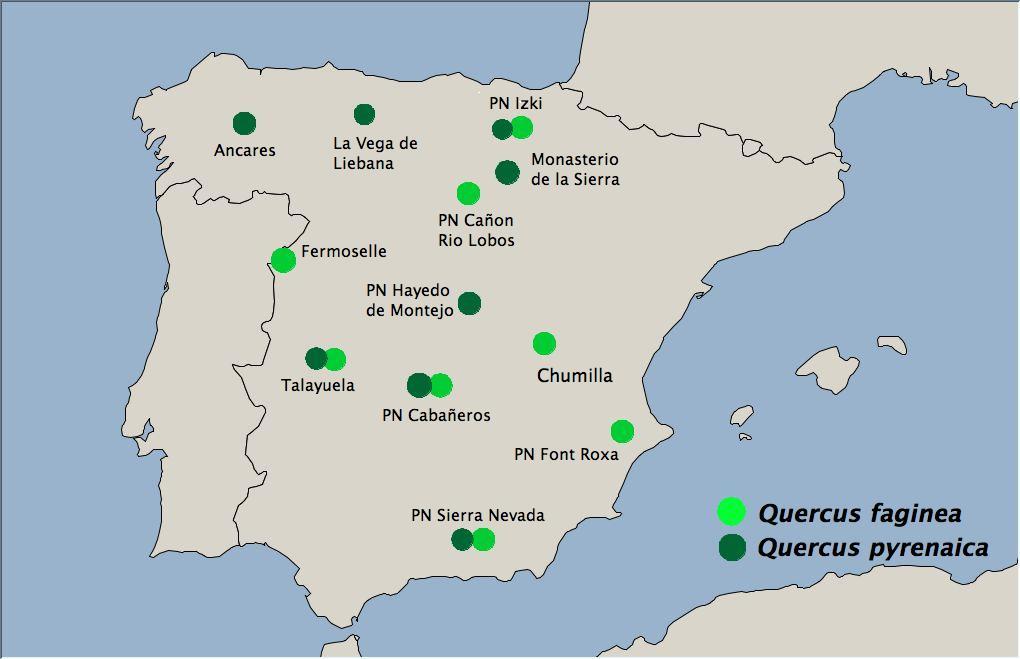

Supplement: Additional file 3 — Map of the populations location of Q. faginea and Q; pyrenaica used for the diversity analyses. [file 1471-2229-12-153-S3.jpeg]

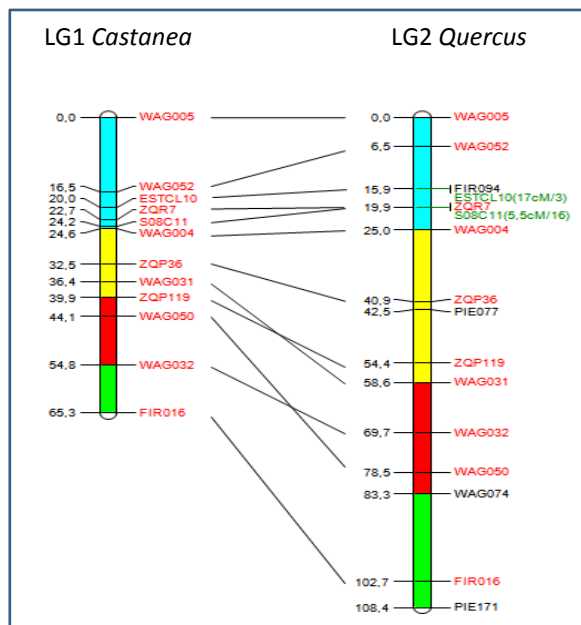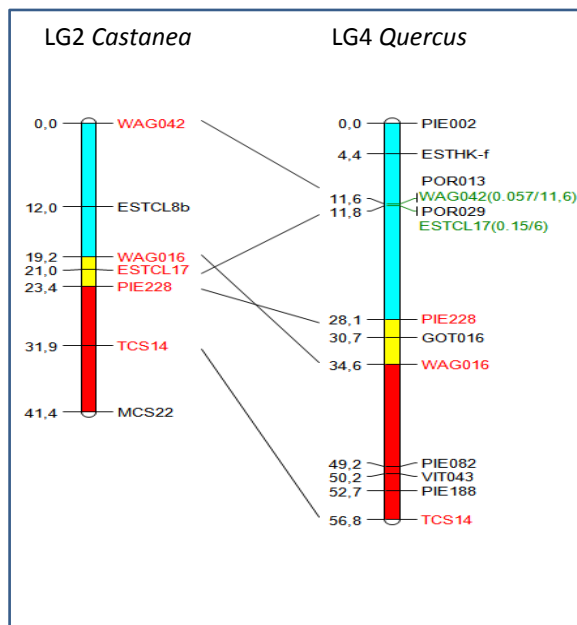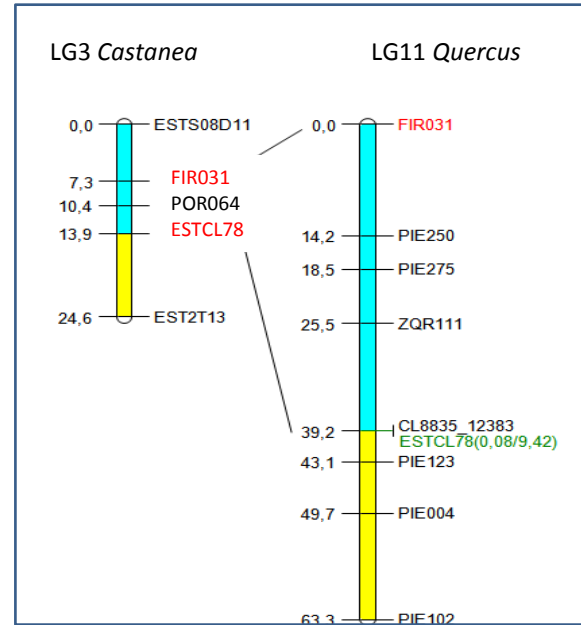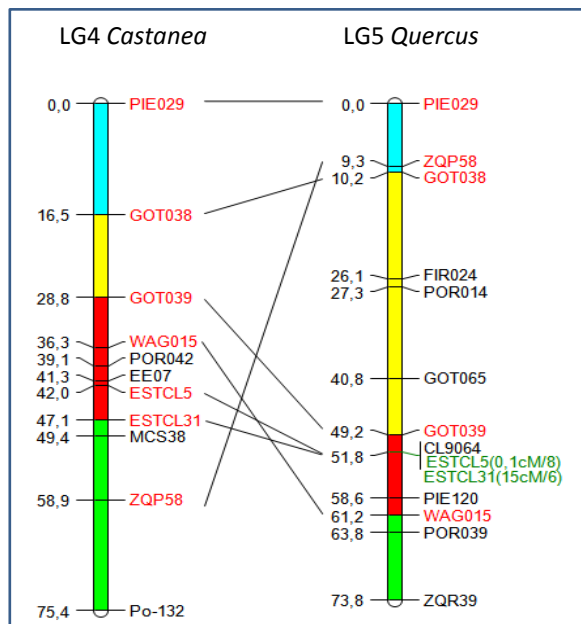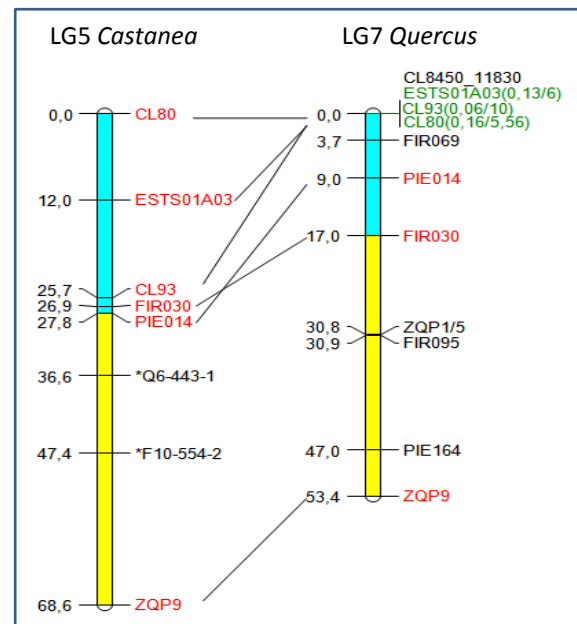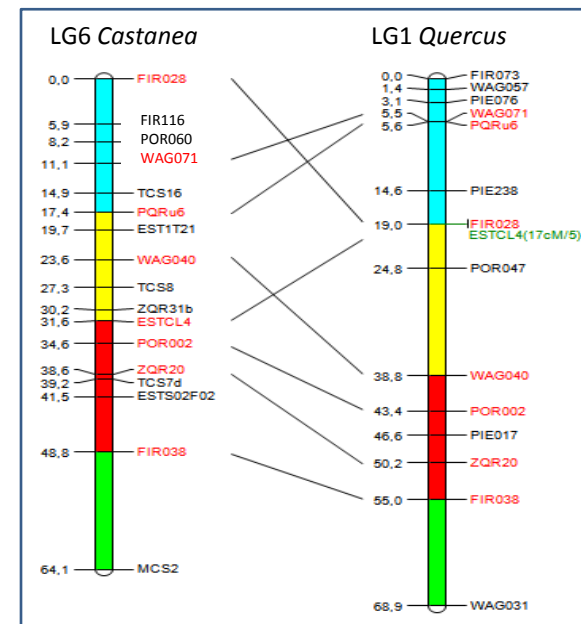

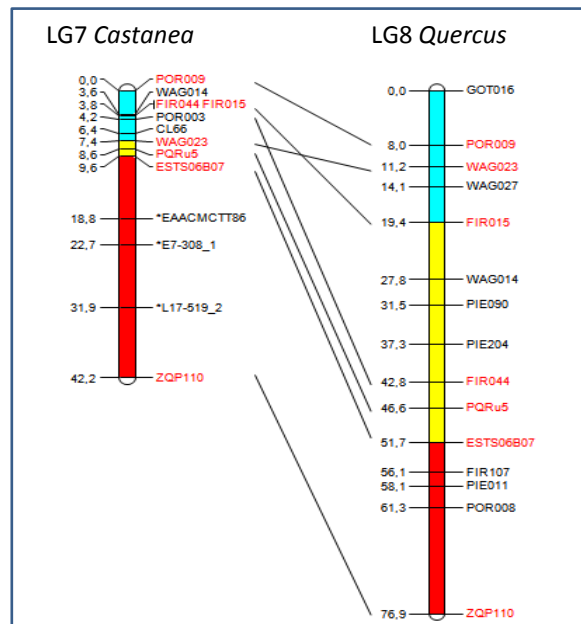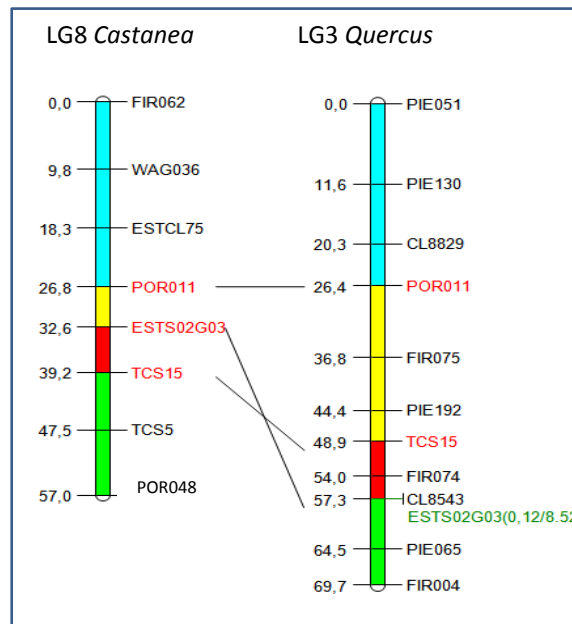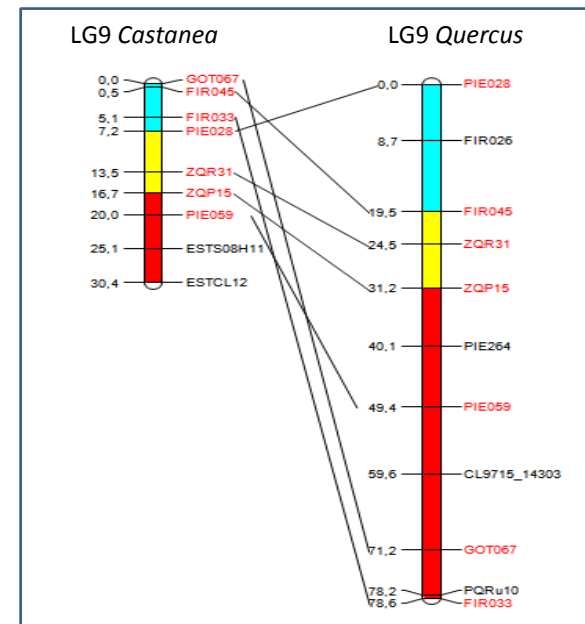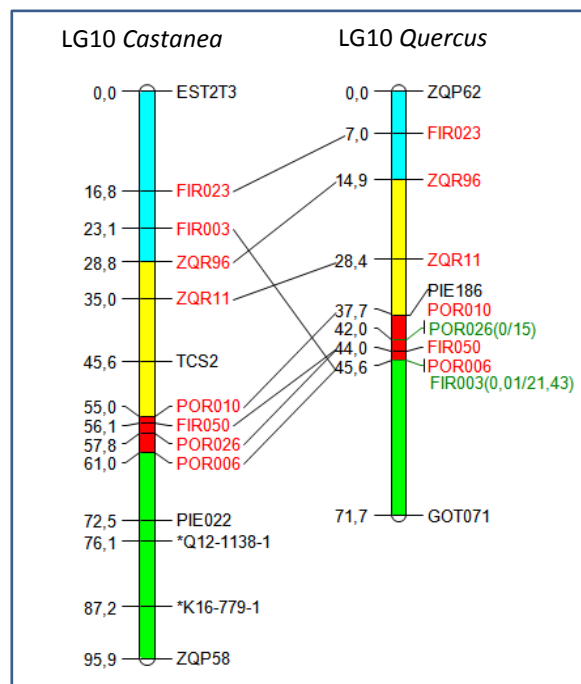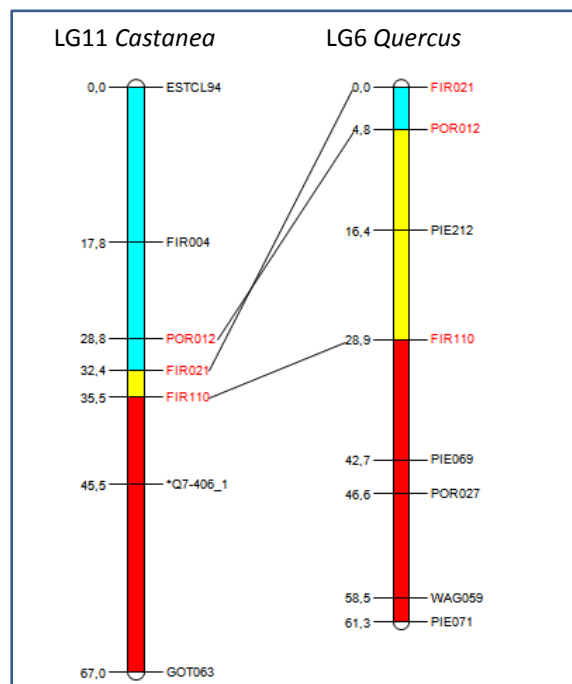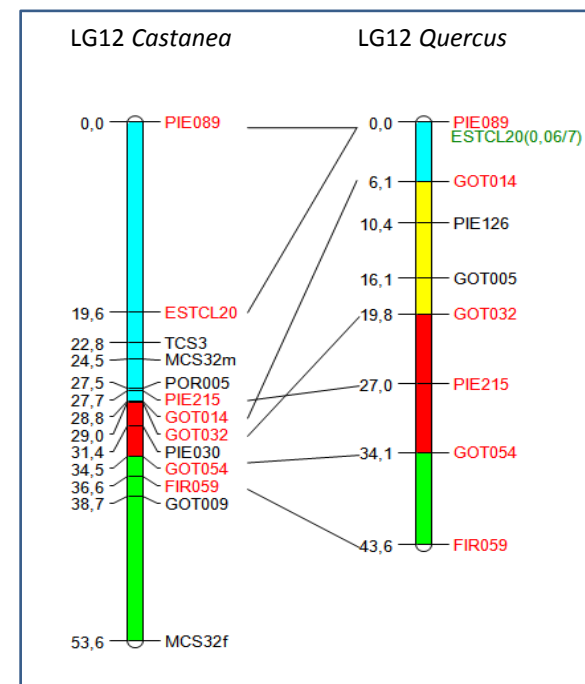

Supplement: Additional file 11 — Linkage groups homology between Castanea and Quercus. [file 1471-2229-12-153-S11.pdf]
