# Supplementary material for: Comparative mapping in the Fagaceae and beyond with EST-SSRs
Source: BMC Plant Biol. 2012 Aug 29;12:153. doi: 10.1186/1471-2229-12-153 (PMC3493355; doi:10.1186/1471-2229-12-153)
Supplement: Additional file 4 — Description of the SSCP method implemented on a Licor DNA sequencer. [file 1471-2229-12-153-S4.docx]

Description of the SSCP method implemented on a Licor DNA sequencer

The SSCP technique is a method capable of identifying most sequence variations in a single strand of DNA, typically between 150 and 250 nucleotides in length. SSCP was carried out using a [LI-COR DNA sequencer. First, a PCR (PCR1) was realized using 20 ng of genomic DNA, 0.2 µM](http://www.exapro.com/li-cor-dna-sequencer-4000l-1993-pe74690/?counter=1&ce_norms=False&text=Dn&product_category=104&currency_best=EUR) of each primer (Primer Forward containing a M13-extension and primer Reverse without extension), 0.5U of Taq Polymerase (GIBCO BRL), 1X Buffer (Invitrogen), 2mM of MgCl_2_ (Invitrogen) and 0.2 mM of dNTPs (Fermentas). PCR conditions were: i/ a first denaturing step (4 min at 94°C), ii/ 35 cycles comprising denaturing (30 sec at 94°C), hybridization (1 min at 58°C) and elongation (1 min at 72°C), and iii/ final extension (5 min at 72°C). For each PCR product, amplification’ specificity is checked using agarose gel electrophoresis (2%).

A second PCR (PCR2) was realized, using 1.5µl of the first PCR product, 0.04µM of primers (M13 primer as forward primer and the same reverse primer as PCR1), 0.5U of Taq Polymerase, 1X buffer, 2mM of MgCl2 and 0.2 mM of dNTPs.

PCR conditions were: i/ denaturation 4min at 94°C, ii/ 30 cycles of denaturing (20sec at 94°C), hybridization (20 sec at 52°C) and elongation (30 sec at 72°C) and iii/ a final elongation of 3 min at 72°C.

1 µl of PCR2 product is combined with 3µl of SSCP loading buffer (95% formamide, 0.05% bromophenol blue, 0.05% xylene blue, 10mM NaOH 2M). Samples were denatured at 94°C for 3 minutes and snap-cooled, and then loaded onto a non-denaturing polyacrylamide gel (5.23ml of MDE gel solution (Lonza, Rockland, USA), 1.27ml TBE 0.6X, 14.4ml H_2_O mQ, 27µl TEMED, and 270 µl APS 10%). The migration was realized at constant temperature and voltage (25°C and 1000V).
