# Supplementary material for: Comparative mapping in the Fagaceae and beyond with EST-SSRs
Source: BMC Plant Biol. 2012 Aug 29;12:153. doi: 10.1186/1471-2229-12-153 (PMC3493355; doi:10.1186/1471-2229-12-153)
Supplement: Additional file 5 — Comparison of Gene Ontology classification between the four sets of sequences containing EST-SSRs (CR: Coding Region, NCR: Non Coding Region, 5’UTR: 5’ Un-Transcribed Region, 3’UTR: 3’ Un-Transcribed Region. The relative frequencies of GO hits for oak sequences are assigned to the GO functional categories (Cellular Compounds, Molecular Function and Biological Process. [file 1471-2229-12-153-S5.docx]

Comparison of Gene Ontology classification between the four sets of sequences containing EST-SSRs (CR: Coding Region, NCR: Non Coding Region, 5’UTR: 5’ Un-Transcribed Region, 3’UTR: 3’ Un-Transcribed Region).

The relative frequencies of GO hits for oak sequences are assigned to the GO functional categories (Cellular Compounds, Molecular Function and Biological Process).

Cellular compounds level2

Molecular Function level2

Biological process level2
