# Supplementary material for: Comparative mapping in the Fagaceae and beyond with EST-SSRs
Source: BMC Plant Biol. 2012 Aug 29;12:153. doi: 10.1186/1471-2229-12-153 (PMC3493355; doi:10.1186/1471-2229-12-153)
Supplement: Additional file 6 — Clustering of Blast2GO categories using the four sets of sequences containing EST (CR: Coding Region, NCR: Non Coding Region, 5’UTR: 5’ Un-Transcribed Region, 3’UTR: 3’ Un-Transcribed Region). [file 1471-2229-12-153-S6.docx]

Clustering of Blast2GO categories using the four sets of sequences containing EST (CR: Coding Region, NCR: Non Coding Region, 5’UTR: 5’ Un-Transcribed Region, 3’UTR: 3’ Un-Transcribed Region)
