# Supplementary material for: Comparative mapping in the Fagaceae and beyond with EST-SSRs
Source: BMC Plant Biol. 2012 Aug 29;12:153. doi: 10.1186/1471-2229-12-153 (PMC3493355; doi:10.1186/1471-2229-12-153)
Supplement: Additional file 7 — Number of loci per LG for the five pedigrees (in () data for two partial linkage group, acc: accessory markers, tot: total number of markers). [file 1471-2229-12-153-S7.docx]

Number of loci per LG for the five pedigrees (in () data for two partial linkage group, acc: accessory markers, tot: total number of markers)

|  | **LG1** | **LG2** | **LG3** | **LG4** | **LG5** | **LG6** | **LG7** | **LG8** | **LG9** | **LG10** | **LG11** | **LG12** | **total** | **mean** |
| --- | --- | --- | --- | --- | --- | --- | --- | --- | --- | --- | --- | --- | --- | --- |
| P1 female |  | (15 +19) | (17+4) |  | (9+7) |  |  |  | (6+4) |  | (8+3) |  |  |  |
| tot | 13 | 34 | 17 | 12 | 16 | 15 | 16 | 15 | 10 | 18 | 11 | 9 | 186 | 15,5 |
| acc | 3 | (3+4) | (3+0) | 4 | (2+1) | 1 | 1 | 3 | (2+0 | 4 | (1+0) | 1 |  |  |
| P1 male |  | (20+14) | (11+4) |  | (6+4) |  |  |  |  |  | (6+5) |  |  |  |
| tot | 13 | 34 | 15 | 6 | 10 | 21 | 15 | 18 | 12 | 13 | 11 | 12 | 180 | 15 |
| acc | 3 | (4+5) | (0+0) | 0 | (0+1) | 5 | 2 | 7 | 1 | 2 | (0+1) | 1 |  |  |
| P2 female |  |  |  | (5+3) |  |  |  |  |  |  |  |  |  |  |
| tot | 12 | 32 | 8 | 8 | 13 | 18 | 7 | 16 | 10 | 7 | 10 | 15 | 156 | 13 |
| acc | 1 | 8 | 1 | (0+0) | 2 | 3 | 1 | 3 | 0 | 0 | 1 | 2 |  |  |
| P2 male |  |  |  |  | (6+5) |  |  |  |  |  |  |  |  |  |
| tot | 8 | 26 | 3 | 5 | 11 | 13 | 6 | 16 | 12 | 12 | ND | 9 | 121 | 11 |
| acc | 1 | 4 | 0 | 2 | (0+0) | 1 | 1 | 4 | 4 | 0 |  | 0 |  |  |
| P3 female |  |  |  |  |  |  |  |  |  |  | (5+5) |  |  |  |
| tot | 18 | 28 | 15 | 8 | 17 | 23 | 11 | 20 | 12 | 11 | 10 | 12 | 185 | 15,4 |
| acc | 6 | 9 | 8 | 3 | 2 | 4 | 2 | 7 | 1 | 1 | (0+1) | 2 |  |  |
| P3 male |  | (16+10) |  | (7+6) | (8+7) |  |  | (10+5) |  |  | (4+4) |  |  |  |
| tot | 19 | 26 | 13 | 13 | 15 | 19 | 10 | 15 | 8 | 14 | 8 | 8 | 168 | 14 |
| acc | 0 | (1+3) | 4 | (4+0) | (1+1) | 5 | 1 | (0+0) | 0 | 4 | (0+0) | 0 |  |  |
| P4 female |  |  |  |  |  |  |  |  |  |  |  |  |  |  |
| acc | 0 | 1 | 3 | 1 | 2 | 0 | 0 | 0 | 0 | 1 | 1 | 0 |  |  |
| P4 male | 1 | 3 | 0 | 0 | 1 | 1 | 2 | 0 | 0 | 0 | 2 | 0 |  |  |
| acc | 9 | 18 | 10 | 3 | 12 | 11 | 5 | 10 | 8 | 10 | 10 | 6 | 112 | 9,33 |
| **mean** | **13** | **26,875** | **11,25** | **7,625** | **12,88** | **16** | **9,75** | **15** | **10,5** | **12** | **9,14** | **9,5** | 153,5 | 12,8 |
| P5 female | 3 | 12 | 3 | 3 | 3 | 4 | 4 | 8 | ND | 5 | 6 | 3 | 54 | 4,5 |
| acc | 0 | 2 | 0 | 0 | 0 | 0 | 1 | 1 |  | 1 | 0 | 0 |  |  |
| P5 male | ND | 24 | ND | ND | ND | ND | 6 | 10 | ND | 4 | 6 | 3 | 52 | 4,33 |
| acc | 1 | 7 |  |  |  |  | 0 | 1 |  | 1 | 0 | 0 |  |  |
