# Supplementary material for: Comparative mapping in the Fagaceae and beyond with EST-SSRs
Source: BMC Plant Biol. 2012 Aug 29;12:153. doi: 10.1186/1471-2229-12-153 (PMC3493355; doi:10.1186/1471-2229-12-153)
Supplement: Additional file 8 — Map length in cM of the ten parental maps (in () are written the values obtained for two partial linkage groups). [file 1471-2229-12-153-S8.docx]

Map length in cM of the ten parental maps (in () are written the values obtained for two partial linkage groups).

|  | \|  \| **LG1** \| **LG2** \| **LG3** \| **LG4** \| **LG5** \| **LG6** \| **LG7** \| **LG8** \| **LG9** \| **LG10** \| **LG11** \| **LG12** \| **tot** \| **mean** \| \| --- \| --- \| --- \| --- \| --- \| --- \| --- \| --- \| --- \| --- \| --- \| --- \| --- \| --- \| --- \| \| P1 female \|  \| (29+109.5) \| (65.5+29.8) \|  \| (29.9+38.3) \|  \|  \|  \| (30.3+13.5) \|  \| (20.5+25.9) \|  \|  \|  \| \|  \| 58.1 \| 138.5 \| 95.3 \| 59.2 \| 68.2 \| 64.3 \| 76.3 \| 67.2 \| 73.6 \| 46.2 \| 46.4 \| 53.3 \| 846.6 \| 70.55 \| \| P1 male \|  \| (61.5+33.7) \| (68.3+32.1) \|  \| (26+3.9) \|  \|  \|  \|  \|  \| (14.4+17.5) \|  \|  \|  \| \|  \| 94.7 \| 92,2 \| 100.4 \| 50.1 \| 29.9 \| 71.6 \| 79.9 \| 74.9 \| 54.5 \| 68.1 \| 31.9 \| 55.9 \| 804.1 \| 67.01 \| \| P2 female \|  \|  \|  \| (33.7+15.3) \|  \|  \|  \|  \|  \|  \|  \|  \|  \|  \| \|  \| 60.7 \| 98 \| 62.4 \| 49 \| 59.5 \| 73.9 \| 61 \| 60.2 \| 38.1 \| 45.6 \| 69.3 \| 71.3 \| 749 \| 62.42 \| \| P2 male \|  \|  \|  \|  \| (29.1+26.5) \|  \|  \|  \|  \|  \|  \|  \|  \|  \| \|  \| 59.5 \| 87.6 \| 20.4 \| 10.7 \| 55.6 \| 73.4 \| 42.7 \| 55 \| 70.5 \| 40.8 \| ND \| 56.7 \| 572.9 \| 52.08 \| \| P3 female \|  \|  \|  \|  \|  \|  \|  \|  \|  \|  \| (13.5+12.2) \|  \|  \|  \| \|  \| 59.1 \| 72.4 \| 60.6 \| 44.8 \| 64.2 \| 57.2 \| 45.7 \| 65.5 \| 69.5 \| 44.6 \| 25.7 \| 30.2 \| 639.5 \| 53.29 \| \| P3 male \|  \| (43.3+36.3) \|  \| (8.9+19.4) \| (23.4+15.8) \|  \|  \| (57.8+19.4) \|  \|  \| (11.2+21.7) \|  \|  \|  \| \|  \| 46.7 \| 79.6 \| 66.5 \| 28.3 \| 39.2 \| 68.7 \| 48.7 \| 77.2 \| 54.9 \| 39.5 \| 32.9 \| 46.2 \| 628.4 \| 52.37 \| \| P4 female \| 57.3 \| 53.2 \| 59.9 \| 47.1 \| 81.2 \| 57.8 \| 49.6 \| 60 \| 62.1 \| 49.1 \| 49.9 \| 51.8 \| 679 \| 56.58 \| \| P4 male \| 68.9 \| 60.7 \| 67.3 \| 22.6 \| 96.6 \| 55.2 \| 20.1 \| 62.7 \| 56.5 \| 41.5 \| 73.3 \| 57.3 \| 682.7 \| 56.89 \| \| **mean** \| **63.1** \| **85.28** \| **66.6** \| **38.98** \| **61.80** \| **65.30** \| **53** \| **65.34** \| **59.96** \| **46.93** \| **47.06** \| **52.84** \| **700.28** \| **58.36** \| \| P5 female \| 10.6 \| 59.2 \| 22.1 \| 34 \| 36.9 \| 40.3 \| 36.1 \| 47.6 \| ND \| 30.5 \| 42.7 \| 32.1 \| 392.1 \| 35.65 \| \| P5 male \| ND \| 120,9 \| ND \| ND \| ND \| ND \| 89.4 \| 83 \| ND \| 37.7 \| 30.4 \| 34.7 \| 396.1 \| 66.02 \| |  |  |  |  |  |  |  |  |  |  |  |  |  |
| --- | --- | --- | --- | --- | --- | --- | --- | --- | --- | --- | --- | --- | --- | --- | --- | --- | --- | --- | --- | --- | --- | --- | --- | --- | --- | --- | --- | --- | --- | --- | --- | --- | --- | --- | --- | --- | --- | --- | --- | --- | --- | --- | --- | --- | --- | --- | --- | --- | --- | --- | --- | --- | --- | --- | --- | --- | --- | --- | --- | --- | --- | --- | --- | --- | --- | --- | --- | --- | --- | --- | --- | --- | --- | --- | --- | --- | --- | --- | --- | --- | --- | --- | --- | --- | --- | --- | --- | --- | --- | --- | --- | --- | --- | --- | --- | --- | --- | --- | --- | --- | --- | --- | --- | --- | --- | --- | --- | --- | --- | --- | --- | --- | --- | --- | --- | --- | --- | --- | --- | --- | --- | --- | --- | --- | --- | --- | --- | --- | --- | --- | --- | --- | --- | --- | --- | --- | --- | --- | --- | --- | --- | --- | --- | --- | --- | --- | --- | --- | --- | --- | --- | --- | --- | --- | --- | --- | --- | --- | --- | --- | --- | --- | --- | --- | --- | --- | --- | --- | --- | --- | --- | --- | --- | --- | --- | --- | --- | --- | --- | --- | --- | --- | --- | --- | --- | --- | --- | --- | --- | --- | --- | --- | --- | --- | --- | --- | --- | --- | --- | --- | --- | --- | --- | --- | --- | --- | --- | --- | --- | --- | --- | --- | --- | --- | --- | --- | --- | --- | --- | --- | --- | --- | --- | --- | --- | --- | --- | --- | --- | --- | --- | --- | --- | --- | --- | --- | --- | --- | --- | --- | --- | --- | --- | --- | --- | --- | --- | --- | --- | --- | --- | --- | --- | --- | --- | --- | --- | --- | --- | --- | --- | --- | --- | --- | --- | --- | --- | --- | --- | --- | --- | --- | --- | --- | --- | --- | --- | --- | --- | --- | --- | --- | --- | --- |
