# Supplementary material for: Comparative mapping in the Fagaceae and beyond with EST-SSRs
Source: BMC Plant Biol. 2012 Aug 29;12:153. doi: 10.1186/1471-2229-12-153 (PMC3493355; doi:10.1186/1471-2229-12-153)
Supplement: Additional file 9 — Estimation of genome length in cM for LOD score ranging from 3 to 5. [file 1471-2229-12-153-S9.docx]

Estimation of genome length in cM for LOD score ranging from 3 to 5
